# Supplementary material for: Inhibition of RANK signaling in breast cancer induces an anti-tumor immune response orchestrated by CD8+ T cells
Source: Nat Commun. 2020 Dec 10;11:6335. doi: 10.1038/s41467-020-20138-8 (PMC7728758; doi:10.1038/s41467-020-20138-8)
Supplement: Supplementary file 1 — Supplementary Information [file 41467_2020_20138_MOESM1_ESM.pdf]

Supplementary Information

Inhibition of RANK signaling in breast cancer induces an anti-tumor immune response orchestrated by CD8+ T cells.

Clara Gómez-Aleza[#], Bastien Nguyen[#], Guillermo Yoldi[#], *et al.*

Supplementary Figures S1-S6

Supplementary Figure S1

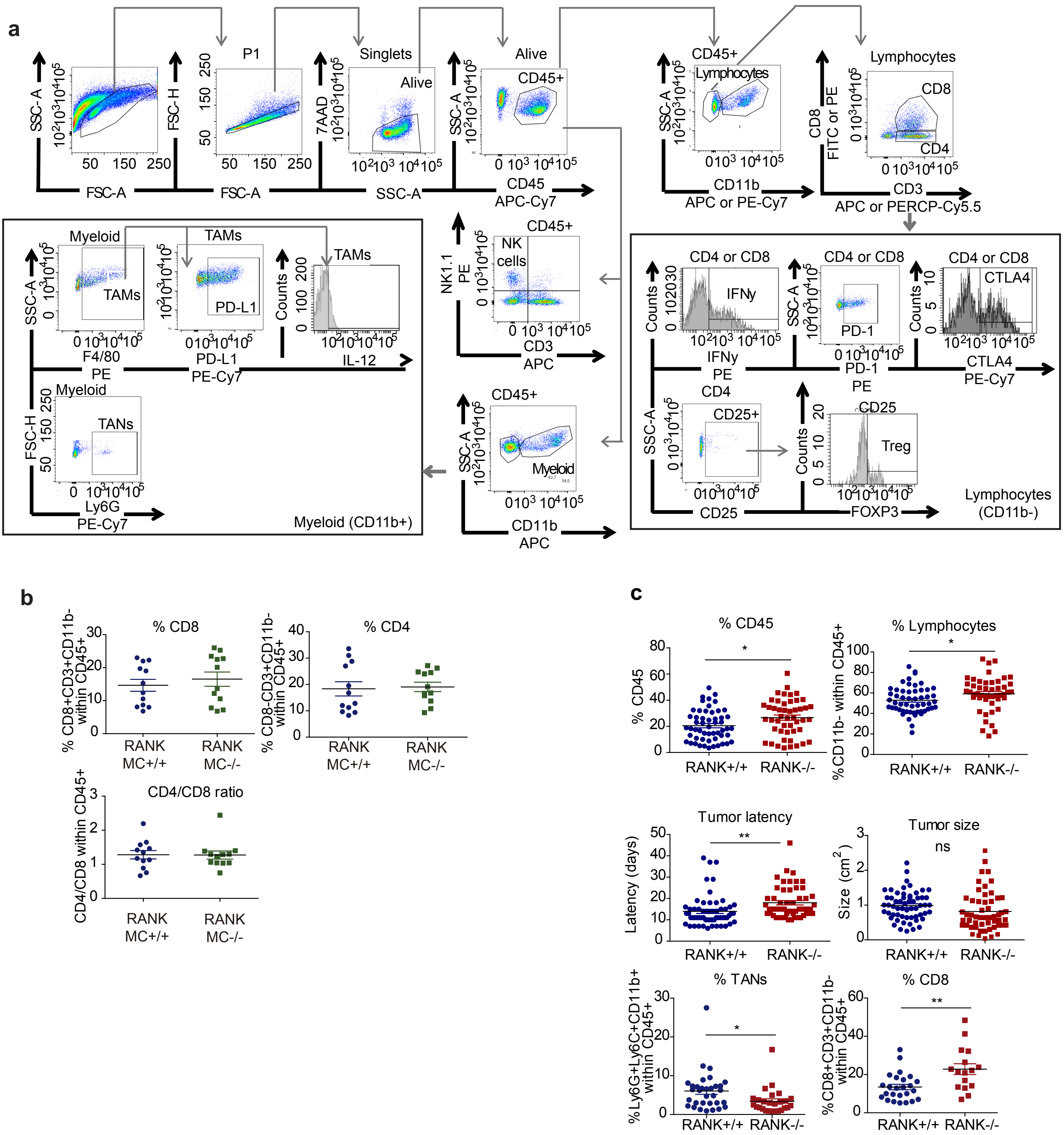

Supplementary Figure S1. Flow Cytometry gating strategy and data from the different immune populations infiltrating mouse tumor transplants.

a. Gating strategy for tumor immune populations. Debris is excluded with FSC-SSC; and single, live, $CD45^+$ cells are selected. $CD11b^-$ cells are gated to enrich in lymphocytes and T cells are gated using CD3. $CD8^+$ T cells are differentiated from $CD4^+$ T cells based on CD8 expression. INFγ, CTLA4 and PD-1 are gated within $CD4^+$ and $CD8^+$ T cells. Tregs are identified by gating $CD25^+$ and $FOXP3^+$ cells within $CD4^+$ T cells. Myeloid cells are enriched by gating the $CD11b^+$ population. TAMs are identified by F4/80+ staining and TANs by $Ly6G^+$ staining. $PDL1^+$ and $IL12^+$ populations are gated within TAMs. NK cells are gated in the $CD45^+$ population as $NK1.1^+CD3^-$ cells.

b. Graphs showing the percentages of tumor-infiltrating $CD8^+$ T cells ($CD8^+$ $CD3^+$ $CD11b^-$ within $CD45^+$), $CD4^+$ T cells ($CD8^-$ $CD3^+$ $CD11b^-$ within $CD45^+$), and CD4/CD8 ratio in $RANK^{+/+}$ tumor transplants in RANK $MC^{-/-}$ and RANK $MC^{+/+}$ mice. Each dot represents one tumor transplant (n=12). Mean +/- SEM is shown.

c. Graphs showing the percentage of leukocytes $CD45^+$ (n= 54 $RANK^{+/+}$ tumors, n=48 $RANK^{-/-}$ tumors; * p = 0.019), lymphocytes (n= 54 $RANK^{+/+}$ tumors, n=48 $RANK^{-/-}$ tumors, $CD11b^-$ within $CD45^+$; * p = 0.0367), TANs (n= 32 $RANK^{+/+}$ tumors, n=26 $RANK^{-/-}$ tumors; $Ly6C^+Ly6G^+CD11b^+$ within $CD45^+$, * p = 0.0224) and $CD8^+$ T cells (n= 25 $RANK^{+/+}$ tumors, n=16 $RANK^{-/-}$ tumors $CD8^+CD3^+CD11b^-$ within $CD45^+$, ** p = 0.0024) in whole-tumor transplants of $RANK^{+/+}$ and $RANK^{-/-}$ in syngeneic C57BL/6 mice. Tumor size at the time of analyses (p = 0.076) and tumor latency (** p = 0.0048) is shown (n= 54 $RANK^{+/+}$ tumors, n=48 $RANK^{-/-}$ tumors). Each dot represents one tumor transplant derived from 12-13 different primary tumors. Means, SEMs and t test two-tailed p values are shown.

Supplementary Figure S2

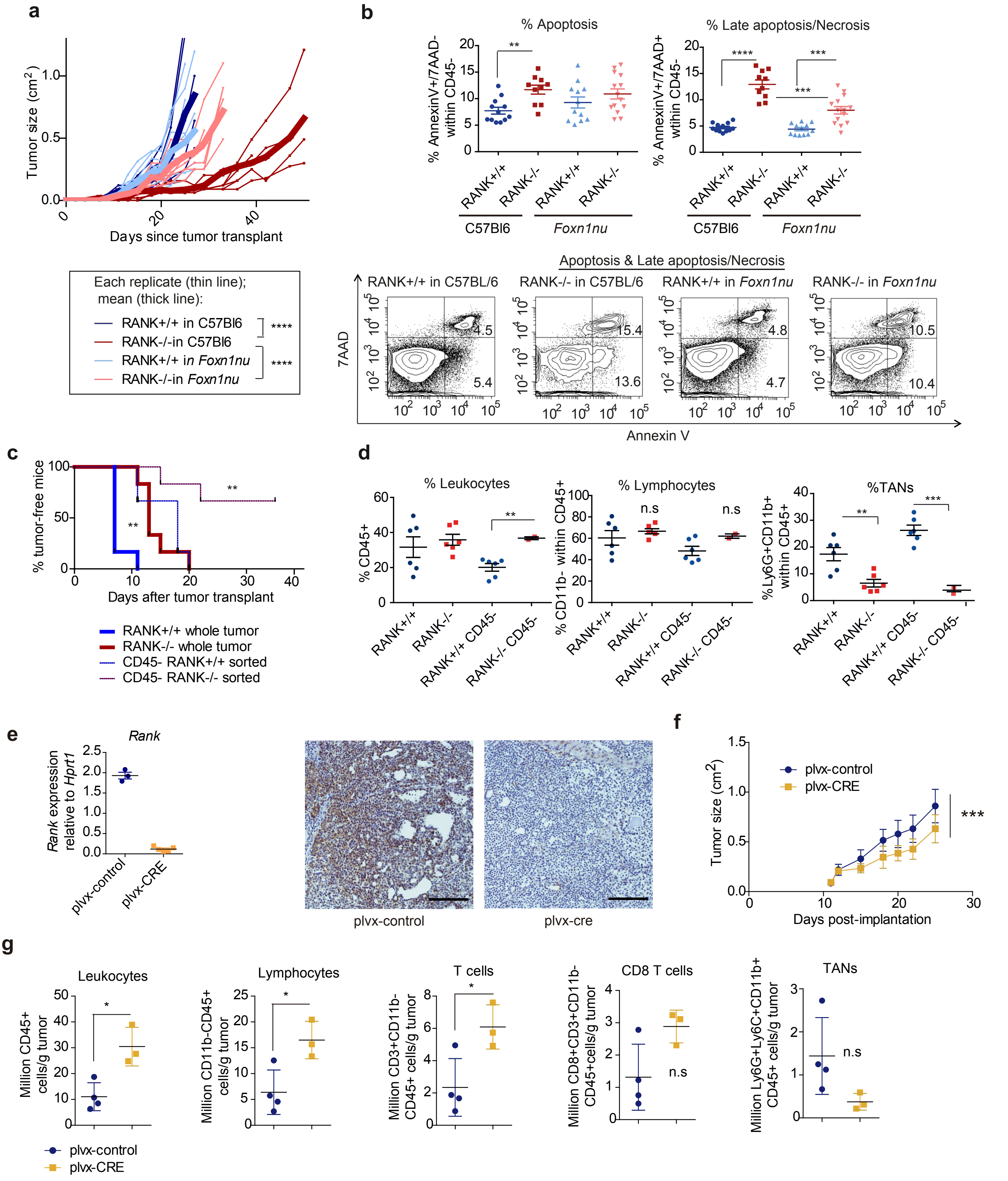

Supplementary Figure S2. Differences in tumor growth and immune infiltration are confirmed upon transplantation of FACs-sorted RANK$^{+/+}$ and RANK$^{-/-}$ cells and upon RANK deletion in an alternative PyMT RANK floxed mouse model.

a. Tumor growth curves after tumor transplantation of RANK$^{+/+}$ and RANK$^{-/-}$ whole tumor cells in syngeneic C57BL/6 (n=6) and *Foxn1^{nu}* (n=8) mice. Thin curves represent each tumor, thick curves represent the mean of all the tumors in each arm.[##].

b. Graphs showing the percentages of apoptotic (AnnexinV^{+}7AAD^{-}, ** p = 0.0011) and necrotic (AnnexinV^{+}7AAD^{+}; **** p < 0.0001, *** p = 0.002) tumor cells in RANK$^{+/+}$ (n = 12) or RANK$^{-/-}$ tumor transplants (n=10 in C57BL/6 or n=14 in *Foxn1^{nu}* hosts) in syngeneic C57BL/6 and *Foxn1^{nu}* mice (n = 10-14 tumors analyzed at endpoint (>0.2 cm^2)). Two different primary tumors were used. Representative dot plots are shown below[#].

c. Kinetics of palpable tumor onset after tumor transplantation of RANK$^{+/+}$ and RANK$^{-/-}$ whole tumor cells (1,000,000 cells injected) or CD45^{-} sorted cells (300,000 cells injected) in syngeneic C57BL/6 mice (n = 6). Note that only 2/6 RANK$^{-/-}$ tumor -sorted cells were able to give rise to transplants. Means and SEMs are shown. Log-rank test with two-tailed p value (whole tumor **, p = 0.013; sorted cells ** p = 0.085).

d. Graphs showing the percentage of CD45^{+} (p = 0.006), lymphocytes (CD11b^{-} within CD45^{+}) and TANs (Ly6G^{+}CD11b^{+} within CD45^{+}; *** p = 0.0009, ** p = 0.003) in tumors described in panel c (n = 6 except for RANK$^{-/-}$ tumor -sorted cells: n=2, see S2c)[#].

e. *Rank* mRNA levels determined by RT-PCR and IHC on PyMT; RANK$^{fl/fl}$ tumors infected with either control or pLVX-cre lentivirus. Scale = 100 µm. (n=3 control and n=4 pLVX-cre-infected tumors). Mean +/- SEM is shown.

f. Growth curves from orthotopically transplanted PyMT; RANK$^{fl/fl}$ tumors infected with control or pLVX-cre lentivirus (n = 6). Mean and SEM are shown (***, p =0.0002)[##].

g. Graphs showing the total number per gram of tumor of leukocytes ($CD45^+$, * $p = 0.01$), lymphocytes ($CD11b^-$ within $CD45^+$, * $p = 0.022$), T cells ($CD3^+$ $CD11b^-$ within $CD45^+$, * $p = 0.03$), $CD8^+$ T cells ($CD8^+$ $CD3^+$ $CD11b^-$ within $CD45^+$, $p = 0.061$) and TANs ($Ly6G^+Ly6C^-CD11b^+$ within $CD45^+$, $p = 0.103$) in tumors described in panel f (n=4 control tumors; n=3 pLVX-cre tumors)[#].

[#]Each dot represents one tumor. Mean, SEM and t test two-tailed p values are shown. (*, $p < 0.05$; **, $p < 0.01$; ***, $p < 0.001$; ****, $p < 0.0001$).

[##]Linear regression analysis was performed and a two-tailed p-value was calculated to compare the tumor growth slopes after the specified treatments (***, $p<0.001$; ****, $p < 0.0001$).

Supplementary Figure S3

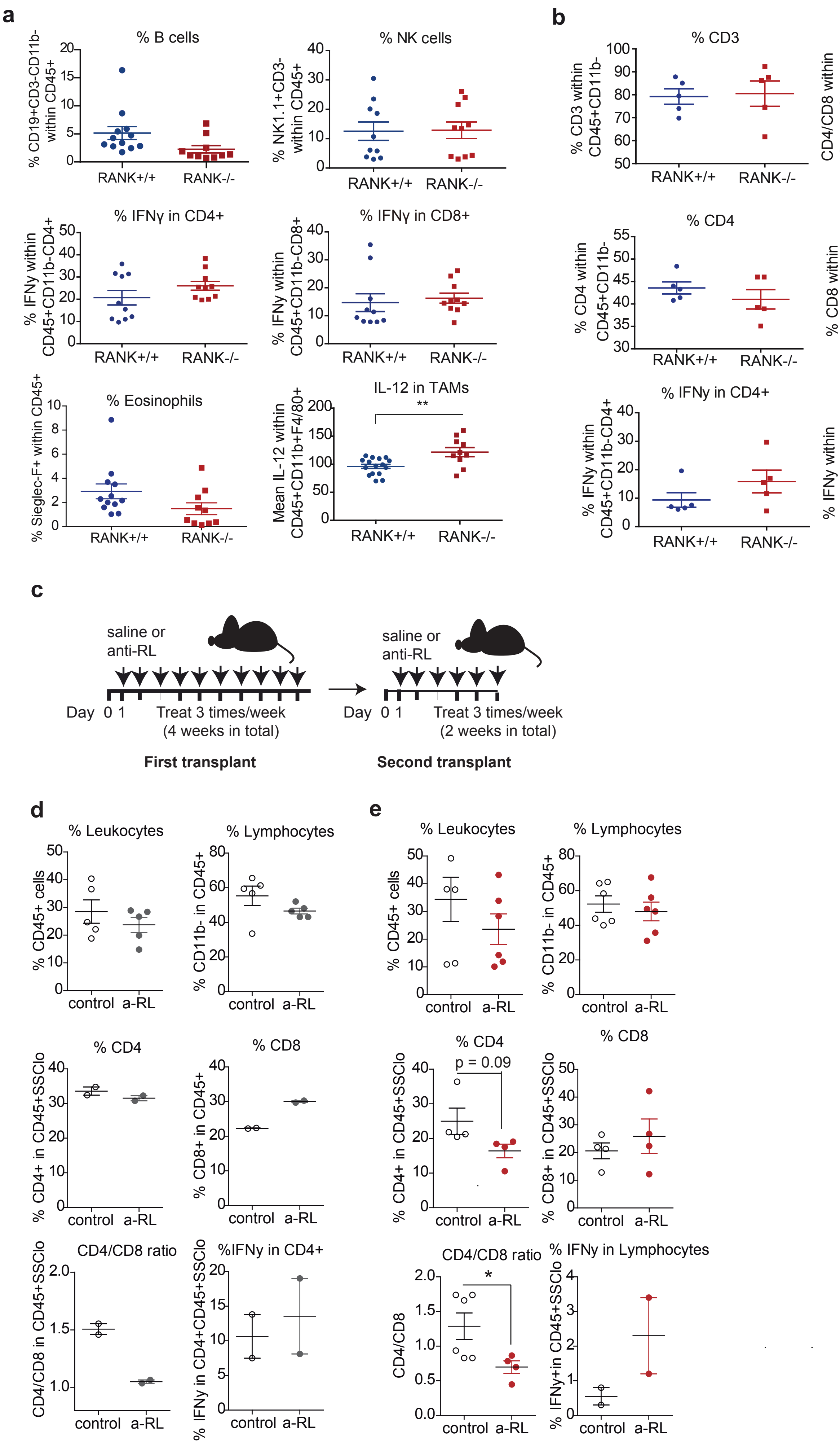

Supplementary Figure S3. RANKL inhibition and immune cell population depletion experiments

a. Graphs showing the percentage of B cells ($CD19^+$ $CD3^-$ $CD11b^-$ within $CD45^+$), NK cells ($NK1.1^+$ $CD3^-$ within $CD45^-$), IFNγ within $CD4^+$ ($CD4^+$ $CD11b^-$ $CD45^+$) and $CD8^+$ ($CD8^+$ $CD11b^-$ $CD45^+$), eosinophils ($SieglecF^+$ $F4/80^+$ $CD11b^+$ within $CD45^+$) and IL-12 within TAMs (IL-12 within $F4/80^+$ $CD11b^+$ $CD45^+$) in whole-tumor transplants of $RANK^{+/+}$ (n = 12) or $RANK^{-/-}$ (n = 10) injected into syngeneic C57BL/6 mice. Each dot represents one tumor. Means and SEMs and t-test two-tailed p values are shown (**, p = 0.0036). Tumor transplants were derived from two representative primary tumors.

b. Graphs showing the percentage of $CD3^+$, $CD4^+$, $CD8^+$, within $CD45^+$ cells, CD4/CD8 ratio and IFNγ expression levels in $CD4^+$ and $CD8^+$ cells in the draining lymph nodes of syngeneic C57BL/6 mice transplanted with $RANK^{+/+}$ and $RANK^{-/-}$ tumor cells (n=5). Each dot represents a pool of two draining lymph nodes from the same mouse. Means and SEMs are indicated. Tumor transplants derived from two representative tumors are considered.

c. Schematic overview of RANK pathway inhibition anti-RL (RANK-Fc) treatments in orthotopic PyMT FvB/NJ tumors. One million cells isolated from a pool of two PyMT carcinomas were orthotopically injected into syngeneic FvB/N mice, which were randomized 1:1 for RANK-Fc (10 mg/kg, three times per week, for 4 weeks) or mock treatment starting 24 h later (first transplant). Tumors were excised 24 h after last treatment analyzed by flow cytometry and cells isolated from each treatment arm were pooled and orthotopically injected into syngeneic FvB/NJ mice (second serial transplant) and treated for 2 weeks with RANK-Fc or mock treatment starting 24 h later. See [11].

d-e. Graphs showing the percentage of leukocytes $CD45^+$ cells (d, n = 5; e, n = 6), lymphocytes ($CD11b^-$ within $CD45^+$; d, n = 5; e, n = 6), $CD4^+$ ($CD8^-$ $CD4^+$ $CD11b^-$ within $CD45^+$; d, n = 2; e, n = 4), $CD8^+$ ($CD4^-$ $CD8^+$ $CD11b^-$ within $CD45^+$; d, n = 2; e, n = 4),

CD4/CD8 ratio (d, n = 2; e, n = 4), and IFN$_{\gamma}$ within CD4^{+} (IFNγ within CD45^{+} CD11b^{-} CD4^{+} CD8^{-}, n = 2) from first (d) or second (e) transplant tumors treated with RANK-Fc or control (n=4) (d) or . Mean, SEM and t test two-tailed p values are shown. (*, p = 0.045).

Supplementary Figure S4

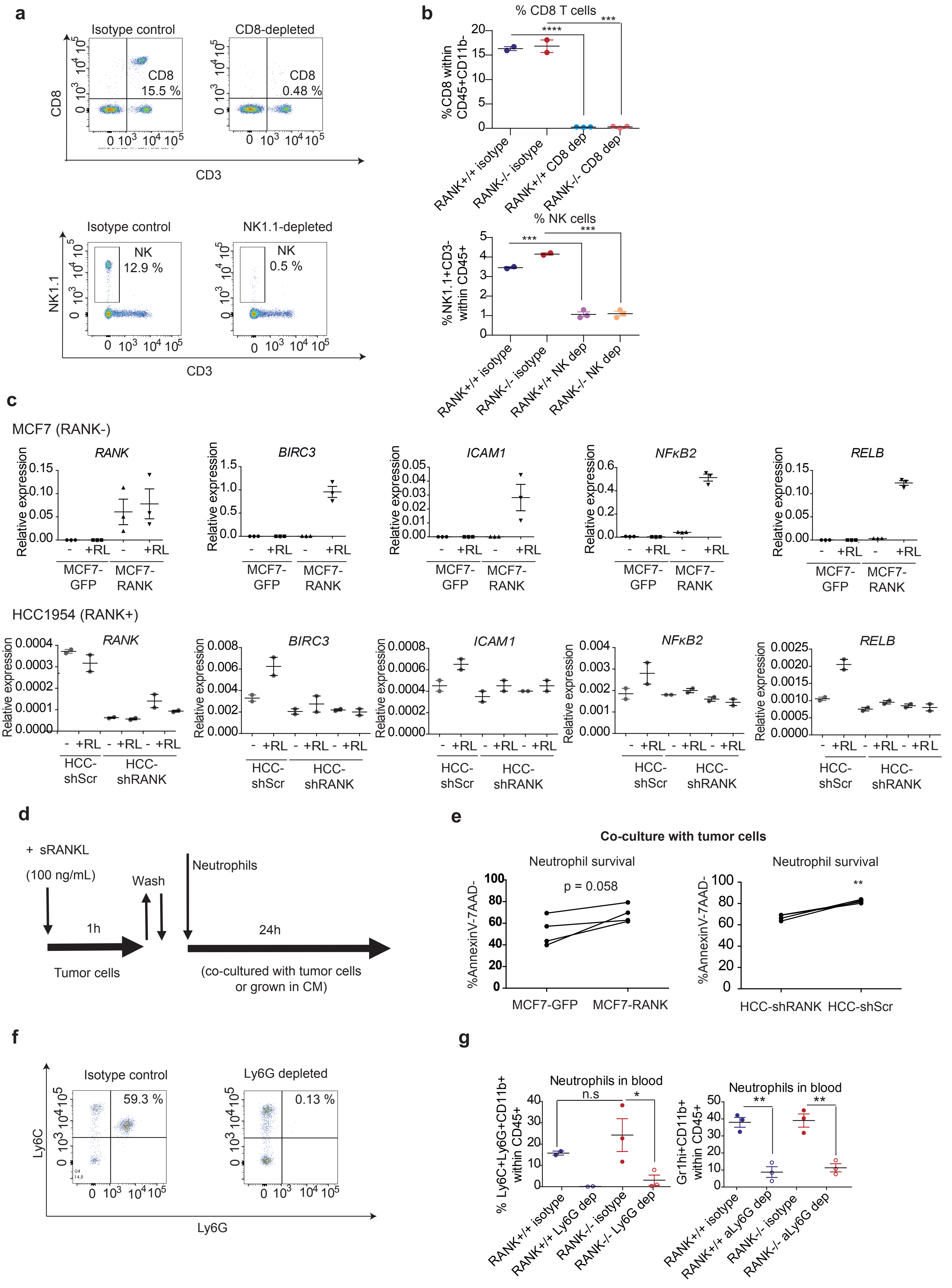

Supplementary Figure S4. RANK pathway activation in breast tumor cells increases survival of immunosuppressive neutrophils.

a-b. Representative dot blots (a) and quantifications (b) showing the percentage of $CD8^+$ ($CD3^+$ $CD8^+$ $CD11b^-$ $CD45^+$; ****, $p < 0.0001$, *** $p = 0.0005$) or NKs ($NK1.1^+$ $CD3^-$; *** $p = 0.0007$ for $RANK^{+/+}$ samples and $p = 0.0003$ for $RANK^{-/-}$ samples) in CD8 depleted (n=3), NK depleted (n=3) and isotype controls (n=3) blood samples from both $RANK^{+/+}$ and $RANK^{-/-}$ tumor transplants 10 days after first treatment with depletion antibodies. Means and SEMs and two-tailed t-test p values are shown.

c. mRNA expression levels of the indicated genes after RL stimulation for 24 h in MCF7-GFP and MCF7-RANK (above, n=3); and HCC1954-shScr and HCC1954-shRANK (below, n=2). Mean +/- SEM is shown.

d. Experimental scheme: human neutrophils isolated from blood of healthy donors are either co-cultured or cultured 1:1 in conditioned medium (CM) from MCF7 (GFP or RANK) or HCC1954 (shScr and shRANK) pre-stimulated with RL for 24 h.

e. Percentage of Annexin-V^- $7AAD^-$ neutrophils (n=4, 2 healthy donors) co-cultured (1:1) for 24 h with the indicated RL-treated tumor cells (MCF7, $p = 0.058$; HCC-1954, ** $p = 0.0047$). Each dot represents neutrophils from technical replicates.

f. Representative dot blots showing the percentage of neutrophils ($Ly6G^+$ $Ly6C^+$ $CD11b^+$ within $CD45^+$) in Ly6G-depleted (n=3) and isotype control (n=3) mouse blood samples.

g Quantification of neutrophils in blood extracted from mice with $RANK^{+/+}$ and $RANK^{-/-}$ tumor transplants 15 days after first treatment with depletion antibody. Left panel shows $Ly6G^+$ $Ly6C^+$ $CD11b^+$ (* $p = 0.05$) and right panel shows $Gr1^{hi}$ $CD11b^+$ within $CD45^+$ (** $p = 0.0024$ for $RANK^{+/+}$ samples and $p = 0.0036$ for $RANK^{-/-}$ samples) Means and SEMs and t-test two-tailed p values are shown.

Supplementary Figure S5

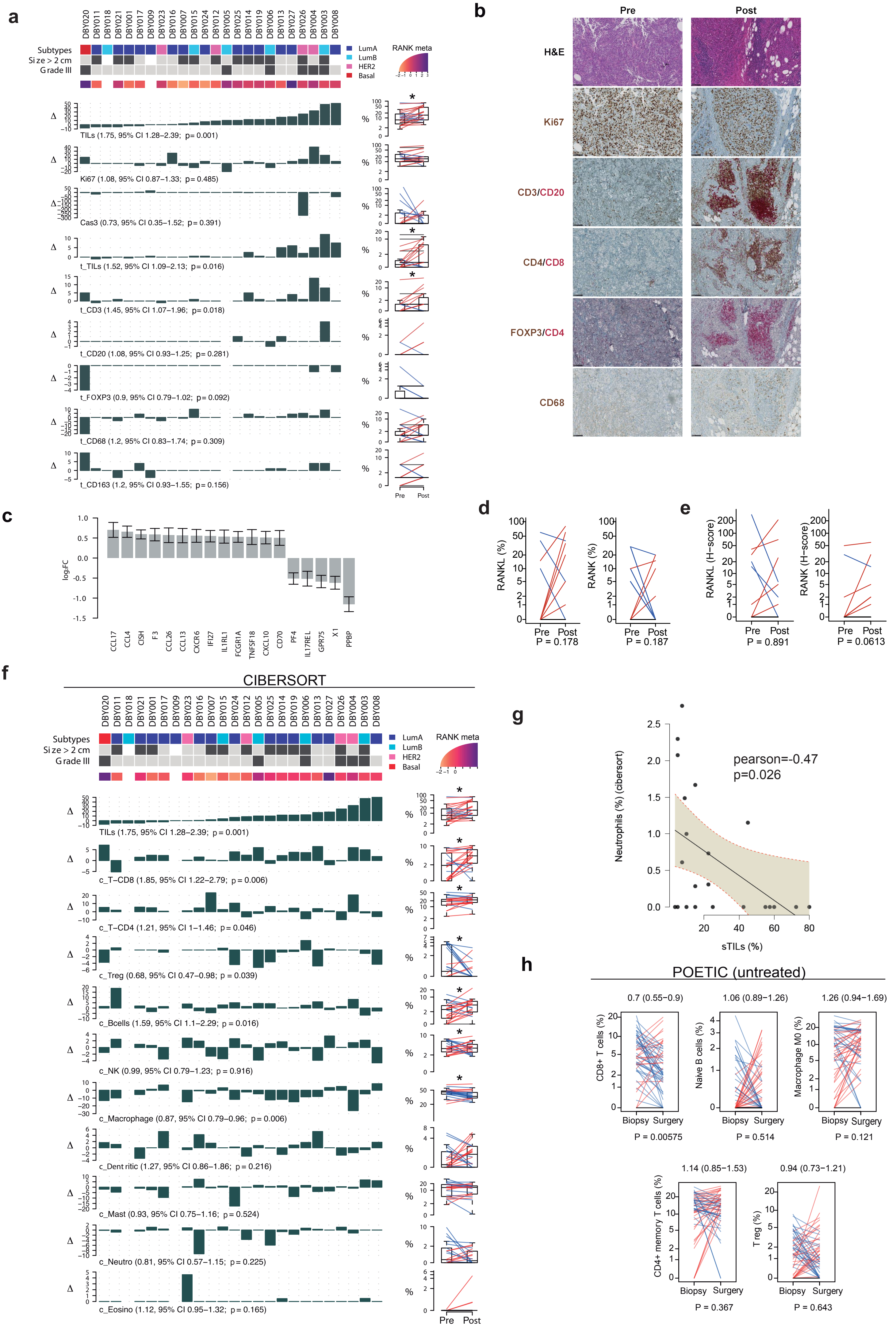

Supplementary Figure S5. Changes in D-BEYOND patient samples after denosumab treatment.

a. Each bar-plot shows the change from baseline (Δ; post- minus pre-treatment values n=24) of each parameter measured using H&E (TILs), IHC (Ki-67 and cleaved caspase-3 tumor cells) and immune intra-tumoral compartment (t_TILs, t_CD3, t_CD20, t_FOXP3, t_CD68, t_CD163) [#, ##]. For each measured parameter, the corresponding boxplot is displayed on the right-hand side. Boxplots display median line, IQR boxes, 1.5*IQR whiskers and data points. Significance assessed by two-tailed paired t-tests.

b. H&E and IHC stainings of pre- and post-treatment tumor sections. One representative patient (DBY003) out of 24 is shown. Scale = 100 μm.

c. Genes differentially expressed between pre- and post-treatment samples (n=24 patients) identified using RNAseq analysis. Boxplots illustrating the $\log_2$ fold change of the differentially expressed genes involved in the GO:0019221 cytokine-mediated signaling pathway. Error bars are plus or minus two times the standard error.

d-e. Change from baseline in protein levels of RANKL and RANK (H-score, n = 24, significance assessed by the sign test) in normal mammary gland adjacent to tumor tissue (d) or in tumor tissue (e). Colored lines indicate increase (red), decrease (blue) or no change (black) relative to baseline. Significance assessed by two-tailed paired t-tests.

f. Each bar-plot shows the change from baseline (Δ; post- minus pre-treatment values) of each nine immune parameters measured using CIBERSORT in 24 patients. Significance assessed by two-tailed paired t-tests. [#, ##]

g. Association between the percentage of neutrophils, as inferred by CIBERSORT, and stromal TIL infiltrations assessed by IHC in surgery samples from D-BEYOND patients. Pearson; P-value derived from the Spearman's correlation, ρ; Spearman's rho. The grey area indicates the 95% confidence interval.

h. Comparison of immune cell fractions as inferred by CIBERSORT, between presurgical biopsies (B) and surgical (S) specimens (n = 57) from the control arm (untreated) of the POETIC study[#].

[#]Geometric mean changes, 95% CIs and p-values are shown below each bar-plot. Colored lines indicate increase (red), decrease (blue) or no change (black) relative to baseline for each patient. All variables were analyzed for all patients, but values for some lines overlap or the indicated population was not detected. Group differences were examined by a two tailed paired t-test (*, $p < 0.05$).

[##]Each bar represents one patient, ordered by increase in stromal TIL levels. For each measured parameter, the corresponding ladder-plot is displayed on the right-hand side. Tumor characteristics and tumor RANK metagene expression at baseline are shown above.

Supplementary Figure S6

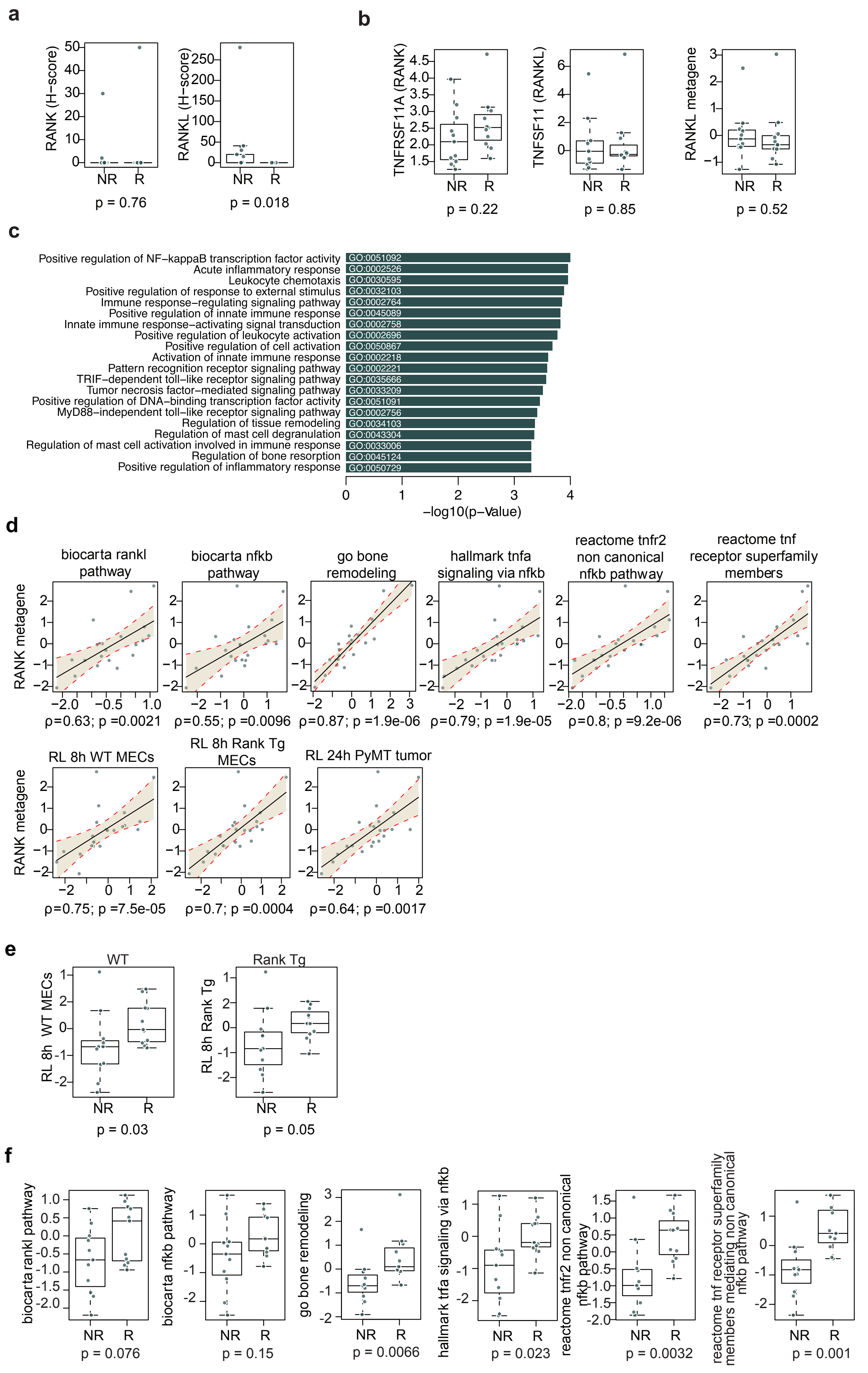

Supplementary Figure S6. RANK metagene as a surrogate marker of RANK activation in biopsy samples and as predictive tool of denosumab-induced immunomodulation.

a. Comparison of RANK and RANKL IHC score in baseline tumor samples between responders (R, n=11) and non-responders (NR, n=11). p, p-values assessed with two tailed Wilcoxon tests. Boxplots display median line, IQR boxes, 1.5*IQR whiskers and data points.

b. Comparison of *TNFRSF11A* (RANK) and *TNFSF11* (RANKL) mRNA expression and RANKL metagene levels in tumor samples between responders (R, n=11) and non-responders (NR, n=11). Boxplots display median line, IQR boxes, 1.5*IQR whiskers and data points. p, p-values assessed with two tailed Mann–Whitney U test.

c. Top 20 most significantly enriched gene ontology (GO) pathways associated with genes included in the RANK metagene. P-values were derived from the two tailed Fisher's exact test.

d. Correlation between RANK metagene and six public signatures related to RANK pathway/NFκB activation from MSigDB (upper) and two signatures derived from the top upregulated genes in WT (left) and RANK-overexpressing (RANK-Tg) 3D cultures of mouse mammary epithelial cells treated with RANKL for 8 h, and RANKL-treated (24 h) PyMT tumor acini-derived gene signature (lower panel). p; p-values assessed with the Spearman's correlation coefficient. The grey area indicates the 95% confidence interval.

e. Comparison of two signatures derived from the top upregulated genes in WT (left) and RANK-overexpressing (RANK-Tg) 3D cultures of mouse mammary epithelial cells treated with RANKL for 8 h between responders (R, n=11) and non-responders (NR, n=11). p, p-values assessed with the two tailed Mann–Whitney U test. Boxplots display median line, IQR boxes, 1.5*IQR whiskers and data points.

f. Comparison of six public signatures related to RANK pathway/NFκB activation from MSigDB between responders (R, n=11) and non-responders (NR, n=11). p, p-values assessed

with the two tailed Mann–Whitney U test. Boxplots display median line, IQR boxes, 1.5*IQR whiskers and data points.

Supplementary Note 1

D-BEYOND Study Protocol

A PRE-OPERATIVE WINDOW STUDY EVALUATING DENOSUMAB, A RANK-LIGAND (RANKL) INHIBITOR AND ITS BIOLOGICAL EFFECTS IN YOUNG PRE-MENOPAUSAL WOMEN DIAGNOSED WITH EARLY BREAST CANCER.

EudraCT number: 2011-006224-21
Sponsor's protocol code number: IJB-BCTL- 20119167

PRINCIPAL INVESTIGATORS/SPONSOR:

Sherene Loi, MD, PhD,
sherene.loi@bordet.be
Christos Sotiriou, MD, PhD,
christos.sotiriou@bordet.be
Martine J Piccart, MD, PhD,
martine.piccart@bordet.be
Hatem A Azim Jr., MD, MSc.
hatem.azim@bordet.be
Breast Cancer Translational Research Laboratory (BCTL)
Jules Bordet Institute
Rue Héger-Bordet 1
Brussels, BELGIUM

STUDY STATISTICIAN:

Stefan Michiels, PhD,
stefan.michiels@bordet.be
Breast Cancer Translational Research Laboratory (BCTL)
Jules Bordet Institute
T:+32 (0) 2 541 3457/3743
F:+32 (0) 2 538 0858

Protocol version [1.0]
Release date: [Nov-2012]

CONFIDENTIAL: Information and data included in this protocol contain trade secrets and privileged or confidential information which is the property of the Jules Bordet Institute. No person is authorized to make it public without the written permission of the director, Jules Bordet Institute. These restrictions on disclosure will apply equally to all future information supplied to you which is indicated as privileged or confidential. This material may be disclosed to and used by your staff and associates as may be necessary to conduct the clinical study.

Additional study contacts:

Medical Enquires

Dr Hatem A Azim Jr., MD or Dr Sherene Loi, MD, PhD

Breast Cancer Translational Research Laboratory (BCTL)

Jules Bordet Institute

T:+32 (0) 2 541 3854/3356/3107

E: hatem.azim@bordet.be; sherene.loi@bordet.be

Translational Research Project Management / Shipping and Transport

Marion Maetens, PhD

Breast Cancer Translational Research Laboratory (BCTL)

Jules Bordet Institute

T:+32 (0) 2 541 7327

F: +32 (0) 2 541 0858

E: marion.maetens@bordet.be

BrEAST Data Centre

Jules Bordet Institute

Study Leader: Céline Faccinetto

T: +32 (0) 2 541 7366

F: +32 (0) 2 541 3477

E : D-Beyond@bordet.be

Study Monitor: Sophie Jamin

T: +32 (0) 2 541 30 85

E: sophie.jamin@bordet.be

Protocol approval – Principal Investigator

The signature below constitutes the approval of this protocol and the attachments, and provides the necessary assurances that this trial will be conducted according to all stipulations of the protocol, including all statements regarding confidentiality, and according to local legal and regulatory requirements and applicable European regulations and ICH guidelines.

Name: Martine J. Piccart

Address: Jules Bordet Institute
Medicine Department
Medical Oncology Clinic
Rue Heger-Bordet, 1
B-1000 Bruxelles
Belgium

Tel: +32 (0) 2 541 32 06

Fax: +32 (0) 2 538 08 58

Signature: .... M. J. Piccart

Date: 20/12/12

Protocol Approval – Principal Investigator

The signature below constitutes the approval of this protocol and the attachments, and provides the necessary assurances that this trial will be conducted according to all stipulations of the protocol, including all statements regarding confidentiality, and according to local legal and regulatory requirements and applicable European regulations and ICH guidelines.

Name: Sherene Loi

Address: Jules Bordet Institute
Breast Cancer Translational Research Laboratory (BCTL)
Rue Heger-Bordet, 1
B-1000 Bruxelles
Belgium

Tel: +32 (0) 2 541 3107

Fax: +32 (0) 2 538 0858

Signature: ....

Date: 17/12/2012

Protocol Approval – Principal Investigator

The signature below constitutes the approval of this protocol and the attachments, and provides the necessary assurances that this trial will be conducted according to all stipulations of the protocol, including all statements regarding confidentiality, and according to local legal and regulatory requirements and applicable European regulations and ICH guidelines.

Name: Christos Sotiriou

Address: Jules Bordet Institute
Breast Cancer Translational Research Laboratory (BCTL)
Rue Heger-Bordet, 1
B-1000 Bruxelles
Belgium

Tel: +32 (0) 2 541 3428

Fax: +32 (0) 2 538 0858

Signature: ....

Date: 18/12/2012

Protocol Approval – Principal Investigator

The signature below constitutes the approval of this protocol and the attachments, and provides the necessary assurances that this trial will be conducted according to all stipulations of the protocol, including all statements regarding confidentiality, and according to local legal and regulatory requirements and applicable European regulations and ICH guidelines.

Name: Hatem A Azim Jr.

Address: Jules Bordet Institute
BrEAST Data Centre
Rue Heger-Bordet, 1
B-1000 Bruxelles
Belgium

Tel: +32 (0) 2 541 3854

Fax: +32 (0) 2 541 3477

Signature: ....

Date: ....17/12/2012....

Protocol approval – Statistician

The signature below constitutes the approval of this protocol and the attachments, and provides the necessary assurances that this trial will be conducted according to all stipulations of the protocol, including all statements regarding confidentiality, and according to local legal and regulatory requirements and applicable European regulations and ICH guidelines.

Name: Stefan Michiels

Address: Jules Bordet Institute
Breast Cancer Translational Research Laboratory (BCTL)
Rue Heger-Bordet, 1
B-1000 Bruxelles
Belgium

Tel: +32 (0) 2 541 3457/3743

Fax: +32 (0) 2 538 0858

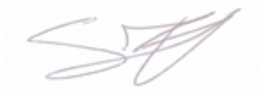

Signature: ....

Date: ....19/12/2012..

Document history

VERSION	MAIN AUTHOR	PROTOCOL VERSION ISSUE DATE	SHORT DESCRIPTION OF MODIFICATIONS
	Hatem A Azim Jr.<br>Sherene Loi<br>Stefan Michiels<br>Marion Maetens<br>Karen Willard-Gallo<br>Evandro de Azambuja<br>Céline Faccinetto<br>Emmanuelle Ceysens<br>Christos Sotiriou<br>Martine J Piccart		

Protocol SYNOPSIS

Study Title	A pre-operative window study evaluating **D**enosumab, a RANK-ligand (RANKL) inhibitor and its **b**iological **e**ffects in **yo**u**n**g pre-menopausal women **d**iagnosed with early breast cancer.
**EudraCT *Number***	2011-006224-21
Sponsor	Jules Bordet Institute
Treatment	Denosumab 120mg subcutaneously Day 1 and 8
Development Phase	Phase IIa
Rationale	RANKL, a TNF-related molecule, has long been reported to be an essential osteoclastic differentiation and activation factor through interaction with its receptor RANK. Denosumab, a monoclonal antibody targeted against RANKL, has been shown to be non-inferior to Zoledronic acid in reducing skeletal-related events in three phase III trials in advanced solid tumors and multiple myeloma<br><br>Lately data has been published that implicates RANK and RANKL as having important roles in other cancer mechanisms. RANKL is known to be involved in inflammation, which influences tumor growth and progression. Using MT2 cells in RAG-/- mammary glands, Tan et al found that most RANKL producing T cells expressed forkhead box P3 (FOXP3), a transcriptional factor produced by T cells which were located next to stromal cells. They demonstrated that targeting RANK-RANKL could be used to prevent the recurrence of metastatic breast disease.<br>Other data have shown that RANK signaling is expressed on the pre-

	malignant and malignant breast neoplasms. Inhibition of RANKL was shown to attenuate breast tumor development both in hormonal dependent and independent models. Furthermore, it has been shown that RANKL signaling is the key mediator of the increase in the mammary stem cell (MaSC) population during pregnancy and lactation and the increase in the MaSC compartment may provide an explanation for the increase in breast cancer incidence seen following pregnancy. At the gene expression level, data from our lab suggest that RANKL expression is associated with breast cancer diagnosed in young women, with the highest expression being observed in pre-menopausal women. This suggests that specific therapeutic approaches will need to be developed in young women in order to improve the poor outcome that has been historically associated with breast cancer diagnosed at a young age. Targeting RANK signaling is one option that could be explored.<br><br>Collectively the data suggests that the effects on RANKL inhibition may go far beyond its osteoclastic actions. Further biological investigation will allow identification and optimization of the breast cancer population that could be helped by targeting RANKL signaling. As such we have designed this biological study to investigate if RANKL treatment can indeed modulate a number of biological processes including RANK signaling and the mammary stem cell subpopulation. We will specifically investigate young women (pre-menopausal) given recent publications of its influence on mammary stem cells and our preliminary data suggesting a role in the breast cancer diagnosed in young women.
Objectives	**Primary:**<br>To determine if a short course of RANKL inhibition with denosumab can induce a decrease in tumor proliferation rates as determined by Ki67 immunohistochemistry (IHC) in newly diagnosed, early stage

<table>
<tr>
<td></td>
<td>
breast cancer in pre-menopausal women.
This will be done by treating women with two doses of denosumab subcutaneously prior to their surgical treatment. Serial tumor and normal tissue biopsies will be taken prior and after treatment (at surgery) to investigate the biological effects of a short course of RANKL inhibition.
Secondary:

To determine the number of absolute Ki67 responders after a short course of denosumab (defined as <2.7% IHC staining in the post treatment tumor biopsy).
To determine the effects of a short course of denosumab on serum C-terminal telepeptide levels (CTX).
To determine the effects of a short course of denosumab on RANK/RANKL gene expression and signaling as assessed by immunohistochemistry (IHC) and gene expression profile in the tumor.
To determine the effect of a short course of denosumab on tumor apoptosis rates using IHC.

➔ To determine these relative changes described above in the surrounding serial normal tissues biopsies.
➔ To determine the relative changes in surrounding normal tissue to that occurring in the serial tumor biopsies.

To determine the effect of a short course of denosumab on modulating the immature mammary epithelial cell populations in the tumor.
To determine the effect of a short course of denosumab on

</td>
</tr>
</table>

	estrogen signaling pathways in the tumor.<br>• To determine the effect of a short course of denosumab on various immune functions, particularly modulation of T regulatory cells in the tumor.<br>• To determine effect of safety profile of denosumab<br><br>➔ To determine these relative changes described above in the surrounding serial normal tissues biopsies.<br><br>➔ To determine the relative changes in surrounding normal tissue to that occurring in the serial tumor biopsies.<br><br>➔ To determine changes according to subgroups defined on PgR status (positive vs. negative)<br><br>➔ To determine these changes according to subgroups defined as RANKL positive and negative in tumor and normal tissue.<br><br>➔ To determine these relative changes described above according to the phase of the menstrual cycle.
Study Design	This is a prospective, single arm phase IIa trial in which patients with early breast cancer will receive pre-operatively two doses of denosumab 120mg subcutaneously one week apart (maximum 12 days) followed by surgery. Surgery will be performed within 10-21 days after the 1st injection. Tumor, normal breast tissue and blood samples will be collected at baseline and at surgery. Post-operative treatment will be at the discretion of the investigator.
Eligibility Criteria	Patients will be eligible for study participation only if they comply with the following inclusion and exclusion criteria:<br><br>**<u>Inclusion criteria:</u>**<br>1) Female gender<br>2) Age ≥ 18 years

	3) Eastern Cooperative Oncology Group (ECOG) performance status 0 or 1<br>4) Premenopausal status defined as the presence of active menstrual cycle or normal menses during the 6 weeks preceding the start of study treatment. Biochemical evidence of phase of menstrual cycle is required (estradiol, FSH and LH). In women previously exposed to hysterectomy, premenopausal levels of estradiol, FSH and LH are required to be eligible<br>5) Non-metastatic operable newly diagnosed primary invasive carcinoma of the breast that is:<br>a) Histologically confirmed<br>b) Primary tumor size greater than 1.5 cm, measured by any of clinical examination, mammography, ultrasound or magnetic resonance imaging<br>c) Any clinical nodal status<br>d) Fully operable and not fixed to chest wall.<br>6) Known HER2 status<br>7) Known estrogen receptor (ER) status and progesterone receptor status (PgR)<br>8) Patient has adequate bone marrow and organ function as shown by:<br>• Absolute neutrophil count (ANC) ≥ 1.5 x 10^9/L<br>• Platelets ≥ 100 x 10^9/L<br>• Hemoglobin (Hgb) ≥ 9.0 g/dL<br>• Serum creatinine ≤ 1.5 x ULN<br>• Total serum bilirubin ≤ 1.5 x ULN (in patients with known Gilbert Syndrome, a total bilirubin ≤ 3.0 x ULN, with direct bilirubin ≤ 1.5 x ULN)<br>• AST and ALT ≤ 1.5 x ULN<br>• Random blood sugar (RBS) ≤ 200 mg/dL or ≤ 11.1 mmol/L<br>• Glycosylated hemoglobin (HbA1c) ≤ 8 %

	9) Albumin-adjusted serum calcium ≥ 8.0 mg/dL (≥ 2.0 mmol/L)<br>10) Women of childbearing potential must agree to use an active local contraception method for the duration of the study and for at least 7 months after the last dose of study treatment<br>11) Patients must accept to take calcium and vitamin D supplementation until the completion of the study treatment<br>12) Signed informed consent form (ICF) for all study procedures according to local regulatory requirements prior to beginning of the study<br>13) Patients must accept to make available tumor and normal tissue samples for submission to central laboratory at the Jules Bordet Institute, Brussels, Belgium, to conduct translational studies as part of this protocol.<br><br>**Exclusion criteria:**<br>1) History of any prior (ipsi and/or contralateral) breast cancer<br>2) Any "clinical" T4 tumor defined by TNM including inflammatory breast cancer<br>3) History of non-breast malignancies within the 5 years prior to study entry (except carcinoma in situ of the cervix, of the colon, melanoma in situ and basal cell and squamous cell carcinomas of the skin)<br>4) Prior or planned systemic anti-cancer therapy before definitive surgery<br>5) Unhealed or planned dental/oral surgery, current or previous osteonecrosis or osteomyelitis of the jaw<br>6) Pregnant or lactating women or women of childbearing potential without a negative serum or urinary pregnancy test within 7 days prior to starting study treatment; irrespective of the method of contraception used

	7) Active Hepatitis-B virus (HBV), Hepatitis-C virus (HCV) or human immunodeficiency virus (HIV) infection<br>8) Known hypersensitivity to denosumab<br>9) Bilateral invasive tumors<br>10) Multifocal/multicentric tumors
Endpoints	**Primary**:<br>• Geometric mean change in tumor Ki67 assessed by immunohistochemistry (IHC) from baseline to prior to surgery<br>**Secondary**:<br>• Absolute Ki67 responders after a short course of denosumab treatment, defined as below 2.7% Ki67 IHC staining in the post treatment tumor biopsy<br>• Decrease in serum C-terminal telepeptide (CTX) levels from baseline to prior to surgery<br>• Change in RANK/RANKL gene expression and signaling as assessed by immunohistochemistry (IHC) and gene expression profile in the tumor<br>• Change in tumor proliferation rates using gene expression (single genes and gene modules, i.e. AURKA, Ki-67) and proliferation-related gene modules, i.e. GGI) in the tumor from baseline to prior to surgery<br>• Change in tumor apoptosis rates as measured using TUNEL and caspase-3 IHC from baseline to prior to surgery<br>• Change in expression levels from genes corresponding to immature mammary epithelial cell populations (MaSCs and luminal progenitors developed by Lim et al; Nature 2009), and in IHC expression of ALDH1, a stem cell marker in the

	tumor.<br><br>• Change in expression levels from single genes related to the estrogen pathways (i.e. ESR1, PgR, BCL2 using both gene expression and IHC) and estrogen-related gene expression modules (i.e. ESR module) in the tumor.<br><br>• Change in expression levels from single genes related to immune pathways using both gene expression and IHC, and in immune-related gene expression modules. This will be done to explore the hypothesis that RANKL can modulate T regulatory cells in the tumor.<br><br>• Change in the quantity of tumor infiltrating lymphocytes as measured by percentage infiltration of surrounding tumor stroma and intra-tumoral on the H&E slide pre and post treatment<br><br>• Safety and tolerability of a short course of denosumab<br><br>➔ The above endpoints will also be characterized in the paired samples of surrounding normal tissue.<br><br>➔ The above endpoints in surrounding normal tissue will be compared to that occurring in the serial tumor tissue biopsies.<br><br>**Exploratory Analyses will also be done according to subgroups defined by:**<br><br>• PgR status (positive vs. negative)<br>• RANKL status (IHC positive vs. negative) in normal breast tissue<br>• RANKL status (IHC positive vs. negative) in infiltrating cells or stroma

	• RANKL status (IHC positive vs. negative) in tumor tissue<br>• RANK status (IHC positive vs. negative) in normal tissue<br>• RANK status (IHC positive vs. negative) in tumor tissue<br>• According to stage of menstrual cycle (luteal or follicular)
Statistical Methods	39 pre-menopausal women will be enrolled to ensure at least 34-paired evaluable samples.<br>The sample size is calculated in order to estimate the geometric mean decrease in percentage stained Ki67 values assessed by IHC in the tumor with a certain precision (i.e. width of a 95% confidence interval around the mean decrease in logged Ki67 values from baseline to prior to surgery, back-transformed to the original scale).<br>We performed an analysis of the data (Miller et al, JCO 2009) of a 14-days letrozole alone window study in order to estimate the expected variability in the change in logged Ki67 IHC in this window study. In that study, the geometric mean decrease in Ki67 values after 14 days of letrozole alone was given by 29% (95% CI 22%-38%) among 51 patients diagnosed with estrogen receptor positive breast cancer. The observed standard deviation of the change in logged Ki67 IHC after 14 days of letrozole alone treatment was 0.98. In order to estimate the mean decrease in Ki67 IHC by denosumab with a 95% confidence interval of a width of 0.66, a total of 34 evaluable patients are needed.<br>The calculation of the gene modules will be done as previously described (Desmedt et al, CCR 2008). For all the endpoints, changes in the values prior to surgery from baseline will be summarized by mean reductions and 95% confidence intervals.<br>Immunohistochemical changes will be assessed using both percentage stained and intensity of staining (Histo-score [Rojo et al,

	CCR 2007]). Pre- and post-treatment values will be compared using the paired Wilcoxon test.<br><br>Exploratory Analysis of Covariance models will be applied to investigate changes in post-treatment values of all the endpoints while adjusting for the pre-treatment baseline values and clinicopathological covariates.<br><br>Safety data will be evaluated using the National Cancer Institute Common Terminology Criteria for Adverse Events (NCI-CTCAE v 4.0).
Translational Research	At baseline: two to four baseline core biopsies (1-2 snap frozen embedded in OCT [optimal cutting temperature] and 1-2 formalin-fixed paraffin embedded) and whole blood/ serum/plasma to be taken on all patients. A sample of normal tissue is required at baseline, (defined as at least 1cm away from tumor, another quadrant or contralateral breast biopsies are accepted as long as repeated in same location at surgery- 1 core frozen, 1 FFPE). A baseline serum sample will also be collected to evaluate CTX levels, which is considered a good surrogate marker for denosumab activity. Estradiol (E2), LH and FSH levels will be measured to establish the phase of the menstrual cycle.<br><br>At surgery: Two to four specimens of residual tumor to be taken at surgery 10-21 days after the first dose of denosumab (1-2 snap frozen embedded in OCT and 1-2 formalin-fixed paraffin embedded) with whole blood/serum/plasma to be taken on all patients. A sample of normal breast tissue should also be taken (1 core frozen, 1 core FFPE) for the biomarker comparisons to normal breast tissue.<br><br>Serial tumor biopsies will be profiled to examine:<br>1/ Gene expression changes of genes representative of RANKL signaling, immature mammary epithelial cell populations, including MaSCs and luminal progenitors. Effects on estrogen-regulated genes, proliferation and immune cells will be examined before and

	after treatment.<br>2/ Tumor infiltrating lymphocytes will be assessed in the samples pre and post therapy using Hematoxylin and Eosin (H&E) FFPE stained sections.<br>3/ Immunohistochemistry will be performed for mammary stem cell markers (i.e. ALDH1), pharmacodynamics effects (RANKL) and relevant immune related genes particularly T regulatory cells.<br>Serial normal tissue biopsies will be evaluated and compared for<br>1/ all of the above endpoints<br>2/ changes in RANK and RANKL IHC pre and post treatment<br>Biological endpoints occurring in tumor will be compared to that occurring in normal tissue and by phase of the menstrual cycle
**Optional Translational Fresh Tissue sub-study**	A sub-study will occur for interested sites, where feasible, where fresh tumor and normal tissue will be collected from the patients participating in the study at the time of surgery for flow cytometry.<br><br>Marker expression for stem cell, epithelial cells, stromal cells and different immune response subpopulations will be quantified in both tissues and blood (the latter for immune markers only).
**Study Duration**	Ideally, four institutions should be able to recruit the desired number of patients within 18 months.

TABLE OF CONTENTS

Additional study contacts: 2

Protocol approval 3

Document history 8

Protocol SYNOPSIS 9

Abbreviations 23

1. Introduction 25

1.1 Background and Rationale 25

1.1.1 RANK-Ligand and bone resorption 25
1.1.2 Targeting RANKL in bone metastases 25
1.1.3 RANKL role in breast cancer: beyond bone metastases 26
1.1.4 Breast cancer in young women 28

1.2 Denosumab 29

1.2.1 Denosumab background 29
1.2.2 Denosumab Preclinical Pharmacology 29
1.2.3 Denosumab Preclinical Pharmacology in Models of Bone Metastasis 30
1.2.4 Denosumab Preclinical Toxicology 31
1.2.5 Denosumab Preclinical Pharmacokinetics and Drug Metabolism 33
1.2.6 Denosumab Clinical Experience 34
1.2.7 Clinical Experience – Pharmacokinetics 35
1.2.8 Clinical Experience – Pharmacodynamics 36
1.2.9 Clinical Experience - Efficacy 36
1.2.10 Safety of proposed study schedule 39
1.2.11 Identified risks 39
1.2.12 Wound complications 41

1.3 Pharmacokinetics and pharmacodynamics of denosumab in humans 41

1.4 Rationale for the proposed trial 42

2. OBJECTIVES 44

2.1 Primary 44

2.2 Key Secondary objectives 45

2.3 Other secondary/exploratory objectives 46

3. STUDY DESIGN 47

3.1 Definition of End of Study 48

3.2 Post study treatment 48

4. STUDY POPULATION 49

4.1 Number of subjects 49

4.2 Eligibility criteria 49

5. STUDY ENDPOINTS 51

5.1 Primary: 51

5.2 Key Secondary 51

5.3 Other secondary/exploratory endpoints 51

5.4 Exploratory Analyses 52

5.5 Translational aspects of the study 53

6. STUDY ASSESSMENTS AND PROCEDURES 54

6.1 Eligibility screening 54

6.2 Treatment period assessments 56
6.3 Pre-surgery visit 56
6.4 Surgery visit........ 56
6.5 After surgery 57
6.6 Safety........ 59
6.6.1 Pregnancy 59
6.6.2 Hypocalcaemia........ 59
6.6.3 Osteonecrosis of the Jaw (ONJ) 60

7. STUDY TREATMENT........ 60
7.1 Investigational product: Denosumab 60
7.1.1 Description........ 60
7.1.2 Dosage form and administration........ 60
7.1.3 Supportive care guidelines 60
7.1.4 Handling and storage........ 60
7.2 DRUG INTERACTIONS 61
7.3 SUBJECT COMPLETION AND WITHDRAWAL 61
7.3.1 Treatment completion........ 61
7.3.2 Subject Completion 61
7.3.3 Subject withdrawal 62
7.3.4 Treatment after the end of the study........ 63
7.3.5 Investigational Product Accountability........ 64

8. SAFETY 64
8.1 Adverse events (AE) and serious adverse events (SAE)........ 64
8.1.1 Definition of a Serious Adverse Event (SAE) 65
8.1.2 Project specific AEs 65
8.1.3 Clinical and laboratory abnormalities as AEs and SAEs 66
8.1.4 Causality of AEs........ 67
8.1.5 Grading of AEs 67
8.1.6 Reporting Procedures for All AEs........ 67
8.1.7 Serious Adverse Events Reporting Procedures........ 68
8.2 Reporting of pregnancy 69
8.3 Death 69

9. DATA ANALYSIS AND STATISTICAL CONSIDERATIONS........ 69
9.1 Analysis sets........ 69
9.1.1 Per-protocol Set........ 69
9.1.2 Safety Set 70
9.2 Primary endpoint and sample size determination........ 70
9.2.1 Accrual rate and study duration 71
9.3 Secondary endpoints........ 71
9.3.1 Key secondary endpoints........ 71
9.3.2 Other secondary endpoints 71
9.4 Subgroup analysis and effect of baseline factors........ 73
9.5 Exploratory analysis 73

10. TUMOR TISSUE HANDLING 74
10.1.1 Biological samples 74
10.1.2 Markers to be assessed 75
10.1.3 Methods for assessment 76
10.1.4 Ki67 Immunohistochemistry 77
10.1.5 Apoptosis markers........ 78

10.1.6 Tumor infiltrating lymphocytes 78
10.1.7 Blood samples 79
10.1.8 Serum CTX levels 79
10.2 Optional Translational Fresh Tissue Sub-study 79
10.3 Tissue samples (please refer to the SPAM) 80
10.3.1 Sample collection 80
10.4 Sample transportation 81
10.4.1 Frozen tissue 81
10.4.2 FFPE 82
10.4.3 Sample storage 82
10.4.4 Translational Fresh Tissue Sub-study 82
11. STUDY CONDUCT AND CONSIDERATIONS 82
11.1 Data handling and record keeping 82
11.1.1 Investigator's file 82
11.1.2 Case Report Form (CRF) 83
11.1.3 Data collection 84
11.1.4 Retention of Documents 84
11.2 Sponsor's Responsibilities 85
11.3 Investigator's Responsibilities 86
11.4 Ethical aspects 87
11.4.1 Local Regulations/Declaration of Helsinki 87
11.4.2 Central and Local ethics committees 87
11.4.3 Informed consent process 87
11.5 Quality Control and Quality Assurance 89
11.5.1 Quality control: on-site monitoring visits 89
11.5.2 Quality assurance 90
11.6 Publication policy 90
11.7 Finance and Insurance 90
11.8 Confidentiality of trial documents and subject records 90
12. References 92
13. APPENDIX 1: ECOG performance status 99
14. APPENDIX 2: Common Terminology Criteria for Adverse Events 100

Abbreviations

AE: Adverse Event

ALT: Alanine Amino Transferase

ANC: Absolute Neutrophil Count

AST: Aspartate Amino Transferase

Cmax: Maximum Serum Drug Concentration

CRF: Case Report Form

CTX: C-terminal telepeptide levels in serum

EC: Ethics Committee

ECOG: Eastern Cooperative Oncology Group

ER: Estrogen Receptor

FFPE: Formalin Fixed Paraffin Embedded

FOXP3: Forkhead box P3

FSH: Follicle-Stimulating Hormone

GCP: Good Clinical Practice

GEP: Gene Expression Analysis

GGI: Genomic Grade Index

GSEA: Gene Set Enrichment Analysis

HbA1c: Glycosylated hemoglobin

HBV: Hepatitis-B Virus

HCV: Hepatitis-C Virus

H&E: Haematoxylin Eosin

Hgb: Hemoglobin

HIV: Human Immunodeficiency Virus

ICF: Informed Consent Form

IHC: Immunohistochemistry

IJB: Jules Bordet Institute

IRB: Institution Review Board

IV: Intravenous

LH: Luteinizing Hormone

MaSC: Mammary Stem Sell

NCI-CTCAE: National Cancer Institute Common Terminology Criteria for Adverse events

OCT: Optimal Cutting Temperature compound

ONJ: Osteonecrosis of the Jaw

OPG: Osteoprotegrin

PD: Pharmacodynamic

PK: Pharmacokinetics

PS: Performance Status

Q4W: Every 4 Weeks

RANK: Receptor Activator of Nuclear Factor Kappa B

RANKL: Receptor Activator of Nuclear Factor Kappa B Ligand

RBS: Random Blood Sugar

SAE: Serious Adverse Event

SC: Subcutaneous

sNTX: N-Telepeptide of Type I Collagen in Serum

SOP: Standard Operating Procedures

SPAM: Study Procedures Administrative Manual

SRE: Skeletal Related Events

TIL: tumor infiltrating lymphocytes

TK: Toxicokinetics

TNF: Tumor Necrosis Factor

TRAIL: TNF-Related Apoptosis-Inducing Ligand

TUNEL: Terminal Deoxynucleotidyl Transferase-mediated Nick-end Labeling Assay

ULN: Upper Limit Normal

uNTx/cr: urinary N-telopeptide normalized to urinary creatinine

Vss: Volume of Distribution at Steady-State

1. INTRODUCTION

1.1 Background and Rationale

1.1.1 RANK-Ligand and bone resorption

The adult skeleton is in a dynamic state of continuous coordinated cycles of bone resorption and bone formation. In response to stressful pressure, there is a continuous interplay between osteoclasts dissolving old bones and osteoblasts laying down new bones in order to maintain the structural bone integrity (Mundy, 1993). The osteoclasts are derived from the haemopoeitic system and under physiological conditions, they are activated to resorb bone and then eventually undergo apoptosis.

The receptor activator of nuclear factor kappa-B ligand (RANKL), a tumor necrosis factor (TNF)-related molecule, has long been reported to be an essential osteoclastic differentiation and activation factor through interaction with its receptor RANK. The RANK-RANKL system has been identified as an essential mediator of osteoclasts formation, function and survival (Khosla, 2001). RANKL binds RANK on the osteoclasts or osteoclast precursors to stimulate or promote differentiation into osteoclasts and activate mature osteoclasts to resorb bone (Teitelbaum and Ross, 2003).

1.1.2 Targeting RANKL in bone metastases

The process of osteolytic metastases depends essentially on the osteoclast-mediated bone resorption and there is a considerable evidence of tumor-induced osteoclastogenesis regardless of the radiological appearance of the disease (i.e. osteolytic or osteoblastic phenotype) (Coleman, 2006; Roodman, 2004). This has promoted the use of bisphosphonates, which are potent inhibitors of osteoclastic bone resorption in the treatment of nearly all types of bone metastases (Aapro et al., 2008). In breast cancer, treatment with bisphosphonates (especially zoledronic acid) results in a significant decrease in the morbidity associated with bone metastases

(Kohno et al., 2005; Wardley et al., 2005). Nevertheless, many patients will continue to develop skeletal-related events (SREs), despite bisphosphonate use.

Targeting RANKL appeared rational as it could arrest the cascade associated with the activation of osteoclasts. Denosumab is a fully human monoclonal IgG2 antibody that specifically binds with high affinity and specificity to the soluble and cell membrane-bound forms of human RANKL (Kostenuik et al., 2009). This results in the inhibition of osteoclastic activity that results in reduced bone resorption, tumor-induced bone destruction, and SREs. Recently, three large phase III trials have shown the non-inferiority of denosumab compared with zoledronic acid established in the primary analyses (Fizazi et al., 2011; Henry et al., 2011; Stopeck et al., 2010). An integrated analysis of all three trials demonstrated superiority of denosumab in delaying or preventing SREs in patients with bone metastases secondary to breast, prostate and other solid tumors ($p<0.0001$) (Lipton et al., 2010). Denosumab significantly reduced the risk of first on-study SRE and subsequent SREs compared with zoledronic acid. However, no difference in disease free or overall survival was observed, although these were investigated as secondary end-points in these trials.

1.1.3 RANKL role in breast cancer: beyond bone metastases

Several recent preclinical studies have highlighted a potential role of RANKL in mediating cancer initiation and progression apart from its aforementioned role in promoting bone metastasis (Asselin-Labat et al., 2010; Gonzalez-Suarez et al., 2010; Joshi et al., 2010; Schramek et al., 2010; Tan et al., 2011).

Mammary epithelial cell development is thought to progress from undifferentiated stem cells into at least two differentiated cell types. Mammary stem cells (MaSCs) have been recently suggested to be controlled partially via a hormone-mediated effect, despite the fact that these cells do not express estrogen and progesterone receptors (Asselin-Labat et al., 2006; Lim et al., 2009). It has been shown that this immature cellular compartment increases up to 11 fold during mid-pregnancy while cells with MaSC phenotype ($CD29^{hi}CD24^{+}$) have reduced capacity in repopulation in ovariectomized mice suggesting that these MaSCs are highly responsive to hormone

stimulation for growth (Asselin-Labat et al., 2010). To elucidate possible paracrine effectors, using unsupervised clustering of genes differently expressed between mammary epithelial cells from ovariectomized and pregnant mice, RANKL emerged as a gene highly expressed during mid-pregnancy but down-regulated in ovariectomized mice. This increase was specific to the luminal cell subsets but in contrast its target receptor, RANK was abundant in the MaSC-enriched cells. In the same experiment, treatment with an anti-RANKL monoclonal antibody impaired the clonogenicity of cells with MaSC features. Taken together, these results suggest that signaling via RANK-RANKL is vital in controlling MaSC populations.

Supporting these results, another study found that RANKL inhibition acts directly on hormone-sensitive mammary epithelium at early stages in tumorigenesis. These investigators found that the hormone progesterone is critical and contributes to increased mammary cancer incidence due to RANKL-dependent proliferative changes in the mammary epithelium (Gonzalez-Suarez et al., 2010). It was also reported that the selective pharmacological inhibition of RANKL attenuated mammary tumor development in hormone- and carcinogen-treated mouse mammary tumor virus-RANK transgenic mice. Such observations were also independently made by another group (Schramek et al., 2010). The latter showed that progestins trigger massive induction of RANKL in the breast epithelial cells and that RANKL inhibition arrest progesterone-driven mammary epithelial cell proliferation and cancers. In addition and as previously noted, RANKL inhibition also impaired the expansion of MaSCs.

Apart from the potential role of RANKL in hormone-driven breast cancer, Tan and co-workers showed a potential role of RANK signaling in tumors that co-express Erbb2 as well (Tan et al., 2011). In this study metastatic spread of Erbb2-transformed carcinoma cells required CD4 (+) CD25 (+) T cells, whose major pro-metastatic function was RANKL production. Most RANKL-producing T cells expressed forkhead box P3 (FOXP3), which is a transcription factor produced by regulatory T cells. The dependence of pulmonary metastasis on T cells was replaceable by exogenous RANKL, which also stimulated pulmonary metastasis of RANK-positive human breast

cancer cells. Together these data suggest that the role of RANK signaling may be critical in maintenance of the normal mammary gland, its growth through pregnancy and lactation and how its deregulation could be critical for breast cancer initiation and growth. Hence, it is critical for this study that normal breast tissue as well as tumor tissue is analyzed, as modulation of the RANK signaling in the normal tissue may provide further insights into denosumab's potential role in breast cancer treatment or prevention.

1.1.4 Breast cancer in young women

Around 7% of patients are diagnosed with breast cancer below the age of 40 (Brinton et al., 2008). These women have poorer survival and higher risk of relapse than their older counterparts (Bharat et al., 2009; El Saghir et al., 2006). Several factors have been linked to the poor prognosis associated with developing breast cancer at a young age. These include large tumor size at diagnosis, higher tumor grade, mitotic rate, lympho-vascular invasion, increased expression of HER2, and lower estrogen and progesterone receptor expression (Anders et al., 2008; Bharat et al., 2009; Cancello et al., 2010). However, even after correction for stage and tumor characteristics, young age at diagnosis remains an independent risk factor for relapse and breast cancer-related death (Adami et al., 1986; Azim et al., 2012; Cancello et al., 2010; Chung et al., 1996; Kim et al., 2011; Kollias et al., 1997) and also an indication for aggressive systemic therapy (Fredholm et al., 2009). At present the biology underlying the poor prognosis of these breast cancers is unknown. Further understanding of the biology would facilitate ongoing efforts to develop specific therapeutic approaches for these women.

In order to help answer this question, a large *in silico* analysis was carried out to examine the genes differentially expressed according to age (Azim et al., 2012). Using a candidate gene approach, a total of 41 genes (including RANKL) and 13 gene sets (including those that represented MaSCs) were investigated. Using a linear regression model adjusted for data source, tumor size, nodal status, histological grade and breast cancer subtype, decreasing age was significantly associated with higher expression of RANKL, MaSCs and luminal progenitors. This was observed in a

training set of 1188 patients ($p<0.0001$) and further confirmed in an independent set of 2334 patients ($p<0.0001$). Of note, these results were independent of the estrogen receptor and HER2 status of the tumor, suggesting this signaling is relevant according to age of cancer diagnosis, rather than specific tumor subtypes. In the same study, the expression of RANKL and MaSCs were highly correlated. These results further support the relation between RANKL and MaSCs and suggest a link to the aggressive biology of breast cancer arising at a young age.

1.2 Denosumab

1.2.1 Denosumab background

Denosumab is a fully human monoclonal IgG2 antibody to RANKL that binds with high affinity (Kd 3 x 10-12 M) and specificity to the soluble and cell membrane-bound forms of human RANKL. Denosumab is highly specific because it binds only to RANKL and not to other members of the TNF family, including TNFα, TNFβ, TNF-related apoptosis-inducing ligand (TRAIL), or CD40 ligand (Elliot et al., 2006; Kostenuik et al., 2009). Denosumab binding prevents the activation of RANK and inhibits the formation, activation, and survival of osteoclasts. As a consequence, bone resorption and cancer-induced bone destruction are reduced.

1.2.2 Denosumab Preclinical Pharmacology

Since the biological activity of denosumab in animals is specific to nonhuman primates, evaluation of genetically engineered (knockout) mice or use of other biological inhibitors of the RANK/RANKL pathway, such as osteoprotegrin (OPG)-Fc and RANK-Fc, were used to evaluate the pharmacodynamic properties of denosumab in rodent models.

In mouse bone metastasis models of estrogen receptor-positive and negative human breast cancer, prostate cancer and non-small cell lung cancer, OPG-Fc reduced osteolytic, osteoblastic, and osteolytic/osteoblastic lesions, delayed formation of de novo bone metastases, and reduced skeletal tumor growth. When OPG-Fc was combined with hormonal therapy (tamoxifen) or chemotherapy (docetaxel) in these

models, there was additive inhibition of skeletal tumor growth in breast, and prostate or lung cancer respectively. In a mouse model of mammary tumor induction, RANK-Fc reduced hormone-induced proliferation in mammary epithelium and delayed tumor formation (XGEVA, 2011).

1.2.3 Denosumab Preclinical Pharmacology in Models of Bone Metastasis

Non-clinical experience in models of bone metastasis is limited to rodent-based experiments using RANKL inhibitors such as OPG-Fc or RANK-Fc, with mechanisms of action that are considered similar to that of denosumab. Mouse models of bone metastasis of human tumor cell lines representing breast, prostate, and lung cancers were used in the preclinical evaluation of the efficacy of RANKL inhibition on reduction of skeletal lesions associated with malignancy and skeletal tumor burden. In a mouse model of estrogen receptor-negative human breast cancer bone metastasis (MDA-231 xenografts), establishment of tumor in the bone marrow was associated with increased osteoclast numbers and significant loss of trabecular bone in untreated mice (Morony et al., 2001). Dosing of OPG-Fc in this model significantly reduced skeletal tumor burden and osteolytic lesions and increased survival in a dose dependent manner. These findings were associated with reduced osteoclast number (Canon et al., 2008). Survival of tumor bearing mice was significantly extended after treatment with OPG-Fc. In addition, pre-treatment of mice with OPG-Fc both delayed and reduced the de novo development of bone metastases, osteolytic lesions, circulating markers of bone resorption and number of osteoclasts. In a similar mouse model of estrogen receptor positive human breast cancer bone metastasis (MCF-7 xenografts), dosing of OPG-Fc significantly reduced skeletal tumor burden, prevented osteolytic lesions, and reduced circulating markers of bone resorption. A similar reduction of skeletal tumor burden and osteolytic lesions was observed following dosing with an estrogen receptor antagonist (tamoxifen) which has an inhibitory effect on estrogen receptor positive cancer cells, and an additive effect on skeletal tumor burden was seen when OPG-Fc was combined with tamoxifen.

Similarly, in a mouse model of human prostate cancer bone metastasis (PC3 xenografts), dosing of OPG-Fc significantly reduced skeletal tumor burden,

significantly reduced osteolytic lesions, and significantly reduced circulating markers of bone resorption. The efficacy was similar to that seen with a high dose of an anti-mitotic anti-cancer agent (docetaxel). The efficacy was additive when OPG-Fc was dosed in combination with docetaxel.

In mouse models of human non-small cell lung cancer bone metastasis (H1299 and H1975 xenografts), dosing with OPG-Fc significantly reduced skeletal tumor burden, prevented osteolytic lesions, significantly reduced circulating markers of bone resorption, and reduced osteoclast number. Upon combination of OPG-Fc and the anti-mitotic cancer agent (docetaxel), the reduction in skeletal tumor was greater than that seen following treatment of either single agent alone.

In summary, RANKL inhibition via OPG-Fc has shown potent effects with respect to inhibiting the development of osteolytic lesions, and the establishment and development of bone metastases correlating with a reduction in circulating markers of bone resorption and osteoclast number. This is consistent with the expected pharmacological activity of RANKL inhibition.

Refer to the current Denosumab Investigator's Brochure for further details.

1.2.4 **Denosumab Preclinical Toxicology**

Standard tests to investigate the genotoxicity potential of denosumab have not been evaluated, since such tests are not relevant for this molecule. However, due to its character it is unlikely that denosumab has any potential for genotoxicity. The carcinogenic potential of denosumab has not been evaluated in long-term animal studies.

In single and repeated dose toxicity studies in cynomolgus monkeys, denosumab doses resulting in 2.7 to 15 times greater systemic exposure than the recommended human dose had no impact on cardiovascular physiology, male or female fertility, or produced specific target organ toxicity.

In preclinical bone quality studies in monkeys on long-term denosumab treatment, decreases in bone turnover were associated with improvement in bone strength and normal bone histology. In male mice genetically engineered to express huRANKL (knock-in mice), which were subjected to a transcortical fracture, denosumab delayed the removal of cartilage and remodeling of the fracture callus compared to control, but biomechanical strength was not adversely affected.

In preclinical studies knockout mice lacking RANK or RANKL had an absence of lactation due to inhibition of mammary gland maturation (lobulo-alveolar gland development during pregnancy) and exhibited impairment of lymph node formation. Neonatal RANK/RANKL knockout mice exhibited decreased body weight, reduced bone growth, altered growth plates and lack of tooth eruption.

Reduced bone growth, altered growth plates and impaired tooth eruption were also seen in studies of neonatal rats administered RANKL inhibitors, and these changes were partially reversible when dosing of RANKL inhibitor was discontinued. Adolescent primates dosed with denosumab at 2.7 and 15 times (10 and 50 mg/kg dose) the clinical exposure had abnormal growth plates. Therefore, treatment with denosumab may impair bone growth in children with open growth plates and may inhibit eruption of dentition (XGEVA, 2011).

Preclinical studies assessing the safety of denosumab included in vitro tissue cross-reactivity studies, assessment of acute effects on the cardiovascular and respiratory system in cynomolgus monkeys, repeated-dose toxicity studies in cynomolgus monkeys up to 1 year in duration, and reproductive and embryo-fetal toxicity studies in cynomolgus monkeys. Tissue cross-reactivity assays were performed to determine the potential of denosumab to bind to tissues other than intended targets. Denosumab did not cross react with any unexpected tissues in panels of human and cynomolgus monkey tissues. Immunoreactivity was observed in lymph nodes, and this reaction was expected because the lymph node is known to express high levels of RANKL (Lacey et al., 1998). Denosumab binding to bone was not noted.

Cardiovascular safety was determined in cynomolgus monkeys and no treatment-related effects were noted on heart rate, mean blood pressure, electrical activity of the heart, or respiratory rate. The initial repeated-dose study in cynomolgus monkeys demonstrated that a dose of 10 mg/kg administered once weekly for 4 weeks was well tolerated. Denosumab was subsequently administered to monkeys once monthly for 1 year and no toxicologically significant effects were observed. Denosumab treatment caused rapid, sustained decreases in bone turnover markers and increases in bone mineral density at doses > 1 mg/kg, which were attributed to the pharmacologic activity of denosumab. Antibodies to denosumab were observed in animals at all doses. Antibody-positive animals had decreased drug levels, and the treatment effect of denosumab on bone markers and bone mineral density was decreased compared with antibody-negative animals.

The genotoxic potential of denosumab has not been evaluated, consistent with guidelines on the preclinical safety evaluation of biotechnology-derived pharmaceuticals (ICH S6). It is not expected that denosumab would interact directly with DNA or other chromosome material. In embryo/fetal development studies, administration of denosumab to female cynomolgus monkeys resulted in increased stillbirths. Based on these findings, denosumab should not be administered to pregnant women, and women of child-bearing potential should continue to use contraception during denosumab therapy and for 7 months after the last dose administered.

Refer to the current denosumab Investigator's Brochure for further details.

1.2.5 Denosumab Preclinical Pharmacokinetics and Drug Metabolism

In mice and rats, species in which denosumab does not bind RANKL, the intravenous (IV) pharmacokinetics (PK) of denosumab were linear over the dose range of approximately 0.1 to 10 mg/kg, with low clearance and a volume of distribution at steady-state (Vss) that indicated a lack of extensive extravascular distribution. Terminal half-life values were 19 and 11 days in mice and rats, respectively. After a single subcutaneous (SC) dose, maximum serum denosumab concentrations

occurred at 72 hours post-dose in both species, and bioavailability was 86% in mice and 56% in rats. In cynomolgus monkeys, the IV PK of denosumab were non-linear over the dose range of 0.0016 to 1 mg/kg but were approximately dose-linear for doses ≥ 1 mg/kg.

At all doses, the Vss indicated a lack of extensive extravascular distribution. The SC PK of denosumab were also nonlinear in monkeys over the dose range of 0.0016 to 1 mg/kg, but were approximately dose-linear between 1 and 3 mg/kg. Based on compartmental modeling, the absolute bioavailability of SC denosumab showed a trend of increasing with dose from 28% to 100%. Modeling of the PK and pharmacodynamic (PD) data (suppression of the bone resorption marker N-telepeptide of type I collagen in serum, sNTx) indicated that denosumab rapidly and potently suppressed bone resorption in monkeys with a 50% effective concentration (EC50) of 464 ng/mL.

The multiple-dose toxicokinetics (TK) of denosumab was evaluated for up to 16 months in cynomolgus monkeys for once weekly or monthly SC doses ranging from 1 to 50 mg/kg. Exposure following the first dose increased approximately dose-proportionally, indicating linear pharmacokinetics over this dose range. No sex difference in denosumab TK was observed. The development of anti-drug antibodies led to decreased exposure relative to exposure after the first dose and to animals that were antibody negative.

Refer to the current denosumab Investigator's Brochure for further details.

1.2.6 Denosumab Clinical Experience

Data are available from 44 clinical studies (27 completed and 17 ongoing) in healthy adults and patients with osteoporosis (approximately 13,500 subjects), bone loss associated with hormone-ablation therapy (approximately 1,900 subjects), rheumatoid arthritis (approximately 200 subjects), advanced cancer (multiple myeloma and advanced malignancies involving bone [approximately 7,800 subjects])

and giant cell tumor of the bone (approximately 260 subjects) collected between June 2001 to November 2010.

Refer to the current denosumab Investigator's Brochure for further details.

1.2.7 Clinical Experience – Pharmacokinetics

Following SC administration, bioavailability was 62% and denosumab displayed non-linear pharmacokinetics with dose over a wide dose range, but approximately dose-proportional increases in exposure for doses of 60 mg (or 1 mg/kg) and higher. The non-linearity is likely due to a saturable target-mediated elimination pathway of importance at low concentrations.

With multiple doses of 120 mg every 4 weeks (Q4W) an approximate 2-fold accumulation in serum denosumab concentrations was observed and steady-state was achieved by 6 months, consistent with time-independent pharmacokinetics. In subjects who discontinued 120 mg every 4 weeks, the mean half-life was 28 days (range 14 to 55 days). A population pharmacokinetic analysis did not indicate clinically significant changes in the systemic exposure of denosumab at steady state with respect to age (18 to 87 years), race/ethnicity (Blacks, Hispanics, Asians and Caucasians explored), gender or solid tumor types. Increasing body weight was associated with decreases in systemic exposure, and vice versa. The alterations were not considered clinically relevant, since PD effects based on bone turnover markers were consistent across a wide range of body weight.

Denosumab is composed solely of amino acids and carbohydrates as native immunoglobulin and is unlikely to be eliminated via hepatic metabolic mechanisms. Its metabolism and elimination are expected to follow the immunoglobulin clearance pathways, resulting in degradation to small peptides and individual amino acids.

1.2.8 **Clinical Experience – Pharmacodynamics**

In phase II clinical studies of patients with advanced malignancies involving bone, subcutaneous (SC) dosing of denosumab (XGEVA™) administered either Q4W or Q12W resulted in a rapid reduction in markers of bone resorption (uNTx/Cr, serum CTX), with median reductions of approximately 80% for uNTx/Cr occurring within 1 week regardless of prior bisphosphonate therapy or baseline uNTx/Cr level. In the phase III clinical trials, median reductions of approximately 80% were maintained in uNTx/Cr after 3 months of treatment in 2075 XGEVA-treated advanced cancer patients naïve to IV bisphosphonate.

Refer to the current XGEVA Summary of Product Characteristics or XGEVA Investigators Brochure for further details.

1.2.9 **Clinical Experience - Efficacy**

1.2.9.1 Efficacy in Subjects with Bone Metastases From Solid Tumors

Efficacy and safety of 120 mg XGEVA SC Q4W or 4 mg zoledronic acid (dose adjusted for reduced renal function) IV Q4W were compared in three randomized, double blind, active controlled studies, in IV-bisphosphonate naïve patients with advanced malignancies involving bone: adults with breast cancer (study 1), other solid tumors or multiple myeloma (study 2), and castrate resistant prostate cancer (study 3) (Fizazi et al., 2011; Henry et al., 2011; Stopeck et al., 2010). Patients with prior history of ONJ or osteomyelitis of the jaw, an active dental or jaw condition requiring oral surgery, non-healed dental/oral surgery, or any planned invasive dental procedure, were not eligible for inclusion in these studies. The primary and secondary endpoints evaluated the occurrence of one or more skeletal related events (SREs). XGEVA reduced the risk of developing a SRE, and developing multiple SREs (first and subsequent) in patients with bone metastases from solid tumors.

1.2.9.2 Efficacy in Subjects with Advanced Malignancies Involving Bone

Denosumab was superior to zoledronic acid in delaying or preventing SREs in subjects with advanced breast cancer and bone metastases in Study 20050136 (Stopeck et al., 2010), in subjects with hormone refractory (castrate-resistant) prostate cancer and bone metastases in Study 20050103 (Fizazi et al., 2011), and in the integrated analysis of the 3 pivotal SRE studies (Lipton et al., 2010). Denosumab was not inferior to zoledronic acid in delaying or preventing SREs in subjects with other solid tumors (excluding breast and prostate cancer) and bone metastases (including subjects with multiple myeloma) in Study 20050244 (Henry et al., 2011).

The hazard ratios were almost identical among individual studies for the risk reduction in the time to first on-study SREs (0.82, 0.84, and 0.82 in Studies 20050136, 20050103, and 20050244, respectively). In addition, the risk was significantly reduced for time to first-and-subsequent on-study SRE in subjects with breast and prostate cancer (0.77 and 0.82 in Studies 20050136 and 20050103, respectively). Furthermore, results from the integrated analyses from the 3 pivotal phase 3 SRE studies demonstrated that denosumab was superior to zoledronic acid for time to first on study SRE (hazard ratio of 0.83 [95% Cl: 0.76, 0.90]; $p < 0.0001$) and time to first and subsequent on study SRE (rate ratio of 0.82 [95% Cl: 0.75, 0.89]; $p < 0.0001$) in subjects with advanced malignancies involving bone (Lipton et al., 2010).

Consistency of denosumab effects was observed across studies, not only for the composite SRE endpoint, but also for the components of the composite endpoint. The integrated analysis demonstrated that denosumab reduced the risk of radiation to bone (hazard ratio of 0.77 [95% Cl: 0.69, 0.87]; $p < 0.0001$) and pathological fracture (hazard ratio of 0.86 [95% Cl: 0.76, 0.96]; $p = 0.0093$), the most frequently occurring types of SREs, compared with zoledronic acid (Lipton et al., 2010). Furthermore, homogeneity testing for time to first on-study SRE in the integrated analysis and in each study showed no evidence of inconsistent effect across the 4 SRE components (pathological fracture, radiation to bone, surgery to bone, and

spinal cord compression) ($p \geq 0.4775$). Denosumab also reduced the risk of developing SRE or malignant hypercalcaemia compared with zoledronic acid by 17% ($p < 0.0001$) (Lipton et al., 2010). In addition, denosumab reduced the risk of a symptomatic SRE (defined as centrally confirmed pathological fractures and spinal cord compressions that were considered symptomatic by the investigator, as well as all surgery to bone and all radiation to bone) compared with zoledronic acid by 21% ($p < 0.0001$). The results across subgroups demonstrate that the 120 mg Q4W regimen is effective across a broad population of advanced cancer patients, irrespective of bone lesion type (osteoblastic, osteolytic, and mixed [osteoblastic/ osteolytic]) and irrespective of the primary tumor type.

1.2.9.3 Clinical Experience - Safety

The safety of denosumab was evaluated in 5,931 patients with advanced malignancies involving bone and is derived from active-controlled, clinical trials examining the efficacy and safety of denosumab versus zoledronic acid in preventing the occurrence of skeletal related events. The adverse reactions are presented in the table below.

Adverse reactions reported in three phase III and one phase II active-controlled clinical studies in patients with advanced malignancies involving bone

**MedDRA system organ class**	**Frequency Category**	**Adverse reactions**
Infections and infestations	uncommon	cellulitis
Immune system disorder	uncommon	Drug hypersensitivity
Metabolism and nutrition disorders	common	Hypocalcaemia
		Hypophosphataemia
Respiratory, thoracic and mediastinal disorders	Very common	Dyspnea
Gastrointestinal disorders	Very common	Diarrhea
	common	Tooth extraction
Skin and subcutaneous tissues disorders	common	hyperhidrosis
Musculoskeletal and connective tissue disorders	common	Osteonecrosis of the jaw

Refer to the current XGEVA Summary of Product Characteristics for further details.

1.2.10 Safety of proposed study schedule

Denosumab has been studied with a loading dose schedule (120 mg SC day 1 and 8) followed by 120mg SC every month in 37 patients diagnosed with recurrent or unresectable giant-cell tumor (Thomas et al., 2010). In this open label study, the primary endpoint was tumor response, defined as the elimination of at least 90% of giant cells or no radiological progression of the target lesion up to week 25. In this study, 30 of the 35 evaluable patients had a tumor response. Adverse events (AEs) were reported in 33 of the 37 patients; the most common being pain in the extremity (n=7), back pain (n=4) and headache (n=4). Five patients had 3-5 AEs, only one of which was deemed to be possibly treatment related: a grade 3 increase in human chorionic gonadotropin concentration that was not related to pregnancy. Five serious adverse events (SAEs) were reported, though none were deemed to be treatment-related. Serum calcium concentrations corrected for albumin were normal except a transient decrease below 2.0 mmol/l (1.9mmol/l) at week 41 for one patient. All patients were instructed to take daily supplements of calcium 500mg and vitamin D 400IU. Overall, in this study patients continued denosumab for far longer than proposed in this study (30 of 35 patients received study drug for longer than 25 weeks) and the loading schedule was not thought to contribute to any specific AEs. The drug was concluded to have potential for further investigation in this tumor type. In conclusion, the proposed schedule of just two doses in this study is thought unlikely to cause any significant AEs for these women.

1.2.11 Identified risks

1.2.11.1 Hypocalcaemia

The risk of hypocalcaemia was more observed with denosumab compared to zoledronic acid in all three trials (Fizazi et al., 2011; Henry et al., 2011; Stopeck et al., 2010). This occurred despite that daily supplementation with calcium (≥ 500mg) and vitamin D (≥ 400 U) was strongly recommended for all patients. However, hypocalcaemia was mostly mild and transient. It mainly occurred during the first 6 months and it was no associated with clinical sequelae. In patients with breast cancer, 5.5% of patients receiving denosumab developed hypocalcaemia compared

to 3.5% for zoledronic acid (Stopeck et al., 2010). However, no difference was observed in developing grade 3 or 4 hypocalcaemia (1.6% vs. 1.2%). In prostate cancer patients, hypocalcaemia was more frequently observed with denosumab compared to zoledronic acid (13 vs. 6%, $p<0.0001$) with more patients developing grade 3 toxicity (5% vs. 1%) (Fizazi et al., 2011). The same was observed in patients with other solid tumors and multiple myeloma (Henry et al., 2011).

1.2.11.2 Renal toxicity

Denosumab is not known to be associated with renal toxicity as it is mainly eliminated by intracellular catabolism in phagocytes, which is the known clearance mechanism of therapeutic monoclonal antibodies (Tabrizi et al., 2006; Wang et al., 2008). This is unlike zoledronic acid, which causes renal impairment and is not indicated for patients with creatinine clearance lower than 30 mL/min, and must be dose-adjusted if creatinine clearance is lower than 60 mL/min, or withheld to minimize the risk for renal failure if creatinine levels rise during treatment. In the aforementioned studies, patients with renal impairment (i.e. creatinine clearance lower than 30 mL/min) were not eligible. In patients with prostate cancer, around 16% of patients receiving zoledronic acid and 15% of those receiving denosumab developed renal impairment (Fizazi et al., 2011). In metastatic breast cancer, there was a clear trend of a higher risk of renal impairment with zoledronic acid in which creatinine elevation was observed in 4% (vs. 3% with denosumab) and more importantly renal failure was observed in 2.5% (vs. 0.2% with denosumab) (Stopeck et al., 2010). Similar observation was made in patients with other solid tumors and multiple myeloma (Henry et al., 2011).

1.2.11.3 Osteonecrosis of the jaw (ONJ)

ONJ emerged as a relatively rare, yet SAE secondary to the administration of bisphosphonates (Bamias et al., 2005; Durie et al., 2005; Marx, 2003; Migliorati, 2003). The definitive symptom is the exposure of mandibular or maxillary bone through lesion in the gingival that do not heal. Poor oral hygiene, recent dental extractions and prior exposure to chemotherapy were risk factors associated with a higher risk of developing ONJ with bisphosphonates. In two of the three trials, there

was a trend of higher risk of ONJ with denosumab compared to zoledronic acid (1 vs. 2% and 1.4 vs. 2%) (Fizazi et al., 2011; Stopeck et al., 2010). In the third trial, the risk was almost the same (1.1 vs. 1.3%) (Henry et al., 2011). Nevertheless, the absolute risk remains low. However, application of appropriate dental preventive measures can significantly reduce ONJ incidence by nearly 80% (Dimopoulos et al., 2009; Ripamonti et al., 2009). This includes avulsion of parodontopathic teeth with marked tooth mobility, which would be likely avulsed within the subsequent 24 months, correction of treatable parodontal conditions and applying superficial and deep oral hygiene treatments with professional root scaling.

1.2.11.4 Acute phase reaction

Zoledronic acid is known to be associated with acute phase reaction during the first 3 days following drug infusion. This is characterized by low grade fever and flu-like symptoms. These symptoms are particularly bothering and could lead to drug discontinuation in some patients. In all three trials, patients receiving denosumab had a lower risk of developing acute phase reaction. In metastatic breast cancer, out of 1,020 patients receiving denosumab, only 106 (10.4%) developed such symptoms compared to 27.3% of patients receiving zoledronic acid.

1.2.12 **Wound complications**

Wound-related complications including wound infection, delayed wound healing or wound dehiscence have been seldom reported by denosumab, in less than 1% of patients (XGEVA, 2011). Hence no specific precautions need to be made prior to surgical intervention given the pre-operative administration of denosumab.

1.3 Pharmacokinetics and pharmacodynamics of denosumab in humans

The PD profile of denosumab appears similar across all of the subject populations studied, which include healthy postmenopausal women, healthy men ≥ 50 years of age, subjects with advanced cancer and bone metastases including breast cancer, other solid tumors, and subjects with rheumatoid arthritis. A single SC dose of denosumab results in a dose-dependent, rapid (within 12 hours); profound (up to

84%) and sustained (up to 6 months) decrease in bone resorption markers, particularly the uNTX-I/Cr.

The PK of denosumab following IV or SC administration has been studied at doses up to 3 mg/kg or 210 mg in various populations as well. It is not notably affected by age, weight, body mass index, sex, race, or renal function. Following SC administration, denosumab exhibits dose-dependent, nonlinear PK over a wide dose range (as observed for other monoclonal antibodies) (Bekker et al., 2004; Kumagai et al., 2011). However, approximately dose-proportional increases in exposure were observed for doses ≥ 60 mg, consistent with saturable and nonsaturable mechanisms of elimination. For a 60-mg or 120-mg dose, maximum serum denosumab concentrations (Cmax) are typically observed 1 to 4 weeks post-dose; after Cmax, serum denosumab levels decline over a period of 4 to 5 months with a mean half-life of approximately 25 to 30 days. The bioavailability of denosumab is approximately 60% after SC dosing. No accumulation in serum denosumab concentrations was observed with repeated doses of 60 mg every 6 months. After repeated doses of 120 mg Q4W, an approximate 2-fold accumulation was observed, as expected based on denosumab's single-dose pharmacokinetic profile, and steady-state was achieved by 6 months. Denosumab pharmacokinetics did not appear to change with time (up to 4 years exposure).

1.4 Rationale for the proposed trial

Collectively the data suggest that the effects on RANKL inhibition may go far beyond its osteoclastic actions. Further focused biological investigation of the mechanisms of this drug in breast cancer could allow identification and optimization of the breast cancer population that could be helped by targeting RANKL signaling. Pre-clinical evidence strongly supports a crucial role for RANKL in breast cancer initiation and progression through modulation of the growth of the MaSC compartment. However, these pre-clinical hypotheses have yet to be validated in human breast cancer.

A large body of evidence suggests that breast cancer arising at a young age is biologically unique and represented by a higher frequency of the more aggressive

breast cancer subtypes (Anders et al., 2011; Azim et al., 2012; Cancello et al., 2010). In addition, survival remains poorer compared to older counterparts even when comparing patients with apparently similar tumor features. Whilst the biology mechanisms for this are unclear, it seems likely that separate therapeutic approaches may need to be developed to improve the poor outcomes for breast cancer diagnosed in young women (Azim et al., 2012). The link between RANK signaling, its effects on immature mammary epithelial cell compartments and recent data linking the different transformation stages of MaSCs to breast cancer subtypes (Lim et al., 2009) provides a rational to investigate in further detail the effects of RANKL inhibition on breast cancers diagnosed in young women. Moreover, denosumab has been received by many thousands of patients in the setting of clinical trials, so its side effect profile is well documented. The specific schedule proposed here has also been previously studied in humans (Thomas et al., 2010) with no significant alterations in the safety profile reported. Hence, the treatment given in the setting of this study is of little risk to the patient with potentially curable disease with the possibility to understand the other biological effects of denosumab on the tumor. This study will be done in the pre-menopausal women in all breast cancer subtypes with the hypothesis that this may be the population most likely to benefit from RANKL inhibition.

The pre-operative setting offers an ideal opportunity to evaluate the role of RANKL on newly diagnosed breast cancer patients. It allows testing the effect of denosumab on RANKL signaling as well as the MaSC content and other related markers via the collection of tumor and surrounding normal tissue before and after drug administration. However this study differs in that it is a short course (two doses) of denosumab that would be administered with the aim to evaluate how RANKL inhibition affects actual biological parameters of the tumor and normal breast tissue. Two doses, a week apart should not result in a significant delay in surgical intervention. Nevertheless, based on the PK of the drug, it should be enough to assess the effect of denosumab on the biological targets of interest as Thomas and colleagues reported that three doses of the 120mg SC could achieve target concentration levels by day 29. This study could serve as a “proof of concept” that

denosumab can affect important pathways that are important in tumor growth or initiation and may help us understand the potential role denosumab could play in influencing important tumor characteristics. An important characteristic of this study is that the patients will have newly diagnosed tumors which may be relevant should the future setting for denosumab in breast cancer be in breast cancer prevention or adjuvant therapy.

A number of studies have been performed that have serial biopsies taken from women before and after a short course of therapy. Particularly, for estrogen receptor-positive breast cancer, these demonstrate that useful biological information can be achieved from biopsies taken at the two time points roughly 14 days apart (Baselga et al., 2012; Baselga et al., 2009; Dowsett et al., 2011; Miller et al., 2009). Short-term surrogate endpoints taken from biopsies just two weeks apart have also been shown to have prognostic implications for these tumors. Using some of these published data, we will be able to understand the significance of the effect of denosumab on some of important tumor characteristics such as proliferation and estrogen signaling (Miller et al., 2009). Finally, exploratory biological analyses could also be performed to discover other pathways that are modulated by a short course of denosumab inhibition. Given the exploratory nature of this study, all breast cancer subtypes will be included in the premenopausal population of interest.

2. OBJECTIVES

2.1 Primary

To determine if a short course of RANKL inhibition with denosumab can induce a decrease in tumor proliferation rates as determined by Ki67 immunohistochemistry (IHC) in newly diagnosed, early stage breast cancer in pre-menopausal women.

This will be done by treating women with two doses of denosumab subcutaneously prior to their surgical treatment. Biopsies of tumor and surrounding normal tissue

will be taken prior and after treatment (at surgery) to investigate the biological effects of a short course of RANKL inhibition.

2.2 Key Secondary objectives

- To determine the number of absolute Ki67 responders after a short course of denosumab (defined as <2.7% IHC staining in the post treatment tumor biopsy).
- To determine the effects of a short course of denosumab on serum C-terminal telepeptide levels (CTX).
- To determine the effects of a short course of denosumab on RANK/RANKL gene expression and signaling as assessed by immunohistochemistry (IHC) and gene expression profile in the tumor.
- To determine the effect of a short course of denosumab on tumor apoptosis rates using IHC.
- To determine these relative changes described above in surrounding serial normal tissue biopsies
- To compare the relative changes in surrounding normal tissue to that occurring in the serial tumor biopsies

Key secondary objectives of this study will be to confirm adequate target inhibition using a surrogate marker in serum, C-terminal telepeptide (CTX). This will serve to ensure that the drug is inhibiting sufficiently its target of interest. Immunohistochemical assessment of RANK/RANKL protein in the tumor will also be assessed to determine prevalence of expression in the dataset, even though preclinical data does not support a decrease due to the drug alone. Each of the primary and secondary endpoints will also be performed in the surrounding normal tissue obtained at baseline and at surgery and according to phase of the menstrual cycle (follicular or luteal). This is to determine the effects of denosumab on normal tissue components according to phase of the menstrual cycle. The key biological secondary objective is to evaluate if two doses of denosumab can cause a change in tumor apoptosis rates, bearing in mind that apoptosis *in vivo* can be difficult to capture due to its rapid turn-over.

2.3 Other secondary/exploratory objectives

- To determine the effect of a short course of denosumab on modulating the immature mammary epithelial cell populations in the tumor.
- To determine the effect of a short course of denosumab on estrogen signaling pathways in the tumor
- To determine the effect of a short course of denosumab on various immune functions, particularly modulation of T regulatory cells in the tumor.
- To determine effect of safety profile of denosumab
- To determine these relative changes described above in surrounding serial normal tissue biopsies
- To compare the relative changes in surrounding normal tissue to that occurring in the serial tumor biopsies
- To determine relative changes according to subgroups defined on PgR status (positive vs. negative)
- To determine these relative changes according subgroups defined as RANKL positive and negative in tumor and normal tissue
- To determine these relative changes described above according to the phase of the menstrual cycle.

Other exploratory objectives will be to determine the modulation of various biological processes important in breast cancer. These will include the hypothesis that denosumab can affect immature mammary epithelial cell populations (such as mammary stem cells and luminal progenitors). As exploratory analyses, we will also investigate the effects on estrogen -regulated genes using previously published gene modules of the estrogen signaling pathway. RANK signaling is thought also to affect some immune processes, particularly as modulation of T regulatory cells (CD4+/CD25+) has been shown to be due to RANKL production. These will also be investigated in the form of gene modules representing various immune pathways as well as IHC markers. Given that there is a well-established relationship between PgR expression and RANKL expression, the effects of denosumab will be analyzed according to subgroups by PgR and RANK/RANKL status. Similarly, given that the

effects of denosumab in vitro were shown to effect the mammary stem cell compartment, analyses and comparisons will also be performed in normal breast tissue and changes compared to that occurring in the serial tumor tissue biopsies. Phase of the menstrual cycle will be evaluated on its effect on endpoints. The exact markers will be defined in the final statistical plan in collaboration with Amgen.

3. STUDY DESIGN

This is a single arm, multi-centre, open label phase IIa study (Figure below). Eligible patients must be pre-menopausal women with primary invasive breast cancer >1.5 cm in diameter who have not undergone previous treatment for invasive breast cancer or being considered for neoadjuvant therapy. This is a pre-operative "window" trial in which all patients will receive denosumab 120mg subcutaneously for two injections administered one week apart prior to surgical intervention. The second dose of denosumab should be given as close to one week (minimum of 7 days to a maximum of 12 days) from the first dose. Serum calcium levels should be measured prior to the second dose.

Tumor, normal tissue, blood, plasma and serum samples will be collected at baseline (prior to therapy) and following the administration of the second dose of the drug or prior to surgery (if the second dose is not received). Estradiol (E2), LH and FSH levels will be assessed to document menopausal status and phase of the menstrual cycle.

Definitive surgery will be performed within 10-21 days after the first dose of denosumab. The second tumor sample will be taken from the residual surgical specimen (i.e. not required for diagnostic purposes).

Surrounding normal tissue samples (1 frozen core, 1 FFPE) will be taken, at baseline and at surgery (defined as at least 1cm away from tumor margins, another quadrant or contralateral breast biopsies acceptable).

Following surgery, all patients will be treated at the discretion of the investigator.

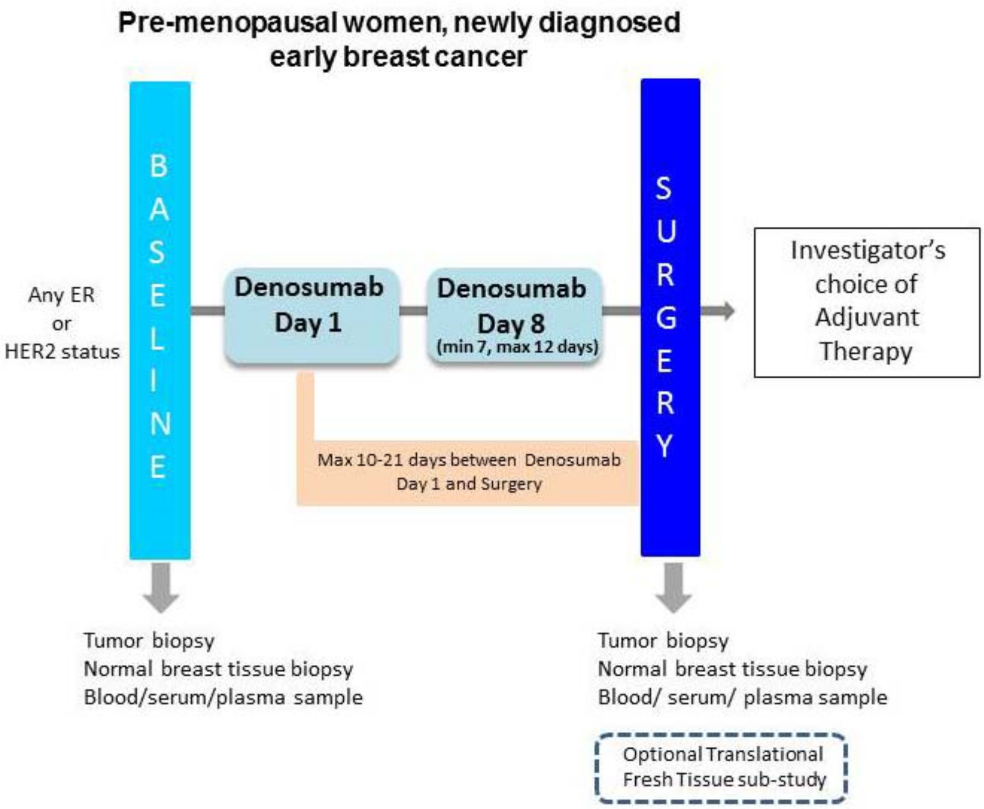

3.1 Definition of End of Study

A subject is considered as having completed the study 3 months after the last dose of study drug. The reporting of the AEs will last for 1 month after the last dose of denosumab except for the project specific AEs (see section 8.1.2) for which the reporting will be extended to 3 months after the last dose of denosumab. All AEs (including the study specific AEs) will be followed up until resolution for a maximum of 3 months following the last dose of denosumab.

The study is considered as completed 3 months after the last enrolled patient has received their last dose of denosumab.

3.2 Post study treatment

Following surgical excision, additional adjuvant systemic therapy (either hormonal and/or chemotherapy) will be at the discretion of the investigator. Post-operative radiotherapy is suggested if breast-conserving surgery has been performed and will be done as per institutional standards.

4. STUDY POPULATION

4.1 Number of subjects

A total of 39 pre-menopausal women with primary breast cancer will be enrolled in this study.

4.2 Eligibility criteria

__Inclusion criteria:__

1) Female gender
2) Age ≥ 18 years
3) Eastern Cooperative Oncology Group (ECOG) performance status 0 or 1
4) Premenopausal status defined as the presence of active menstrual cycle or normal menses during the 6 weeks preceding the start of study treatment. Biochemical evidence of phase of menstrual cycle is required (estradiol, FSH and LH). In women previously exposed to hysterectomy, premenopausal levels of estradiol, FSH and LH are required to be eligible
5) Non metastatic operable newly diagnosed primary invasive carcinoma of the breast that is:
    a. Histologically confirmed
    b. Primary tumor size greater than 1.5 cm, measured by any of clinical examination, mammography, ultrasound or magnetic resonance imaging
    c. Any clinical nodal status
    d. Fully operable and not fixed to chest wall.
6) Known HER2 status
7) Known estrogen receptor status (ER) and progesterone status (PgR)
8) Patient has adequate bone marrow and organ function as shown by:
    - Absolute neutrophil count (ANC) ≥ 1.5 x 10^9/L
    - Platelets ≥ 100 x 10^9/L
    - Hemoglobin (Hgb) ≥ 9.0 g/dL
    - Serum creatinine ≤ 1.5 x ULN

- Total serum bilirubin ≤ 1.5 x ULN (in patients with known Gilbert Syndrome, a total bilirubin ≤ 3.0 x ULN, with direct bilirubin ≤ 1.5 x ULN)
- AST and ALT ≤ 1.5 x ULN
- Random blood sugar (RBS) ≤ 200 mg/dL or ≤ 11.1 mmol/L
- Glycosylated hemoglobin (HbA1c) ≤ 8 %

9) Albumin-adjusted serum calcium ≥ 8.0 mg/dL (≥ 2.0 mmol/L)
10) Women of childbearing potential must agree to use active local contraception method for the duration of the study and for at least 7 months after the last dose of study treatment
11) Patients must accept to take calcium and vitamin D supplementation until the completion of the study treatment
12) Signed informed consent form (ICF) for all study procedures according to local regulatory requirements prior to beginning of the study
13) Patients must accept to make available tumor and normal tissue samples for submission to central laboratory at the Jules Bordet Institute, Brussels, Belgium, to conduct translational studies as part of this protocol.

Exclusion criteria:

1) History of any prior (ipsi and/or contralateral) breast cancer
2) Any “clinical” T4 tumor defined by TNM including inflammatory breast cancer
3) History of non-breast malignancies within the 5 years prior to study entry (except carcinoma in situ of the cervix, of the colon, melanoma in situ and basal cell and squamous cell carcinomas of the skin)
4) Prior or planned systemic anti-cancer therapy before definitive surgery
5) Unhealed or planned dental/oral surgery, current or previous osteonecrosis or osteomyelitis of the jaw
6) Pregnant or lactating women or women of childbearing potential without a negative serum or urinary pregnancy test within 7 days prior to starting study treatment; irrespective of the method of contraception used

7) Active Hepatitis-B virus (HBV), Hepatitis-C virus (HCV) or human immunodeficiency virus (HIV) infection
8) Known hypersensitivity to denosumab
9) Bilateral invasive tumors
10) Multifocal/multicentric tumors

5. STUDY ENDPOINTS

5.1 Primary:

- Geometric mean change in Ki67 response assessed by IHC from baseline to prior to surgery in the tumor

5.2 Key Secondary

- Absolute Ki67 responders after a short course of denosumab treatment, defined as below 2.7% Ki67 IHC staining in the post treatment tumor biopsy
- Decrease in serum C-terminal telepeptide (CTX) levels from baseline to prior to surgery
- Change in RANK/RANKL gene expression and signaling as assessed by immunohistochemistry (IHC) and gene expression profile in the tumor
- Change in tumor proliferation rates using gene expression (single genes and gene modules, i.e. AURKA, Ki-67) and proliferation-related gene modules, i.e. GGI) in the tumor from baseline to prior to surgery
- Change in tumor apoptosis rates as measured using TUNEL and caspase-3 IHC from baseline to prior to surgery

5.3 Other secondary/exploratory endpoints

- Change in expression levels from genes corresponding to immature mammary epithelial cell populations (MaSCs and luminal progenitors developed by Lim et al; Nature 2009), and in IHC expression of ALDH1, a stem cell marker in the tumor.

- Change in expression levels from single genes related to the estrogen pathways (i.e. ESR1, PgR, BCL2 using both gene expression and IHC) and estrogen-related gene expression modules (i.e. ESR module) in the tumor.
- Change in expression levels from single genes related to immune pathways using both gene expression and IHC, and in immune-related gene expression modules. This will be done to explore the hypothesis that RANKL can modulate T regulatory cells in the tumor.
- Change in the quantity of tumor infiltrating lymphocytes as measured by percentage infiltration of surrounding tumor stroma and intra-tumoral on the H&E slide pre and post treatment
- Safety and tolerability of a short course of denosumab
- The above endpoints will also be characterized in the paired samples of surrounding normal tissue.
- The above endpoints in surrounding normal tissue will be compared to that occurring in the serial tumor tissue biopsies.

5.4 Exploratory Analyses

Subgroup analyses will also be performed according to:

- PgR status (positive vs. negative)
- RANKL status (IHC positive vs. negative) in normal breast tissue
- RANKL status (IHC positive vs. negative) in infiltrating cells or stroma
- RANKL status (IHC positive vs. negative) in tumor tissue
- RANK status (IHC positive vs. negative) in normal tissue
- RANK status (IHC positive vs. negative) in tumor tissue
- According to stage of menstrual cycle (luteal or follicular)

5.5 Translational aspects of the study

This study attempts to identify pathways relevant to tumor growth that are modulated secondary to the administration of denosumab in newly diagnosed breast cancers in pre-menopausal women. Tumor tissue collection is therefore mandatory in this study. The genes and pathways of interest will be examined at both the gene expression and the protein level. This will allow us to explore the mechanism of action of denosumab *in vivo* and elucidate its potential role in managing patients developing breast cancer. In addition, this study could help in identifying biomarkers that can predict benefit of the drug. Preclinical studies have identified a potential role for breast cancer prevention for this agent. Therefore, it was felt it was best to evaluate the biological effects of a short-term administration of denosumab in newly diagnosed breast tumors as well as the effects on surrounding normal breast tissue.

To prospectively address this issue, breast cancer and surrounding normal tissue samples will be collected at two time points: at baseline and from the residual tumor material (i.e. not required for diagnostic purposes) collected at the definitive surgical operation to remove the tumor (i.e. before and after drug administration). Paired samples of surrounding normal tissue from the breast are requested at baseline as well as surgery. This is to allow evaluation of biological endpoint changes in normal tissue as well as facilitating comparisons to changes occurring in paired tumor tissue samples. Phase of the menstrual cycle will be documented at baseline.

Whole blood, serum and plasma will also be collected. Serum samples will be used to assess the C-terminal telepeptide (CTX) levels. CTX is a bone resorption marker and is considered a good surrogate marker for denosumab activity (Bekker et al., 2004; Fizazi et al., 2009; Kumagai et al., 2011; Lipton et al., 2007). Whole blood and plasma samples will be collected in case of future circulating markers study or a potential marker of response to denosumab is found.

Furthermore an optional translational sub-study will be performed in sites where the logistics are feasible. Fresh tumor, normal tissue samples and blood will be collected

at the time of surgery to assess cellular subpopulations using flow cytometry. Marker expression for stem cell, epithelial cells, stromal cells and different immune response subpopulations will be quantified in both tissues and blood (the latter for immune markers only).

6. STUDY ASSESSMENTS AND PROCEDURES

All baseline evaluations must be completed prior to treatment initiation. Relevant information will be captured in a case report form (CRF). All other data will be recorded in source documents. A signed informed consent must be obtained before any study specific assessments are initiated. Procedures conducted as part of the subject's routine clinical management (e.g. blood counts) and obtained prior to signing of the informed consent may be utilized for screening purposes provided these procedures are conducted as specified in the protocol and within the protocol-defined time-frames.

All patients enrolled in the trial will be followed, according to the schedule outlined in the schedule of assessment below, from the day of the informed consent signature until 3 months after last dose of denosumab.

6.1 Eligibility screening

All patients must provide written informed consent before any study specific assessments or procedures are performed.

An Eligibility Screening Form (ESF) documenting the patient's fulfillment of the entry criteria is to be completed by the Investigator/designee for all individuals considered for the study. This form will be sent to the sponsor (D-beyond@bordet.be) for verification and approval of the patient inclusion. The following will be obtained at screening within a maximum of 1 month before first study drug administration, unless differently specified:

- Demographic data: date of birth and race, physical examination and vital signs (height in centimeters, body weight in kilograms, ECOG Performance

status, blood pressure, heart rate, respiratory rate and body temperature), menopausal status including the biochemical evidence of the menopausal status (LH, FSH, estradiol levels) and the phase of the menstrual cycle, pregnancy test within 7 days prior to starting study treatment (urine or serum β-HCG), current contraception and history of oral contraception for all the potentially childbearing patients

- Complete medical history including breast cancer history (date of diagnosis and primary tumor characteristics), clinically significant prior diseases and surgical procedures and significant prior or concomitant medications.
- Hematology, hemostatic functions, blood chemistry and serology tests:
  - Hematology: counts of hemoglobin, neutrophils, platelets, leukocytes.
  - Hemostatic functions: PT, PTT, INR.
  - Blood chemistry: AST, ALT, ALP, bilirubin total, direct bilirubin, serum creatinine, albumin-adjusted serum calcium, electrolytes (P-, Mg2+, Na+, K+), albumin, uric acid, random blood sugar, glycosylated hemoglobin (HbA1c)
  - Serology: HBV, HCV, HIV
  - Serum CTX levels
- Radiological exams: at least a mandatory breast radiological examination should be performed (mammography, MRI or ultrasound). The breast imaging must be performed on both breasts, unless a mastectomy has been performed.
- Biological samples: tumor, normal breast tissue and blood samples
  - Tumor samples: 1-2 frozen and 1-2 FFPE cores. In some cases, tissue suitable for molecular studies have been obtained during a routine diagnostic procedure performed prior to study enrolment (in between 10-21 days).
  - Surrounding normal tissue samples: 1 frozen core, 1 FFPE (defined as at least 1 cm away from tumor, another quadrant or contralateral breast biopsies accepted as long as repeated in same location at surgery)

    - Blood samples: 10 ml of blood, serum and plasma for future biomarker evaluation.

6.2 Treatment period assessments

The assessments at visit Day 8 should be performed before the second administration of study drug.

- Physical examination, ECOG performance status and vital signs
- Evaluation of serum FSH, LH and estradiol to determine the phase of the menstrual cycle
- Blood chemistry tests: albumin-adjusted serum calcium. In case of hypocalcaemia grade 3-4, the second dose of denosumab should not be given.
- AEs including SAEs documented according to NCI-CTCAE v 4.0
- Concomitant medications and procedures.

6.3 Pre-surgery visit

The pre-surgery visit should be performed at least 1 day after the second administration of study drug but always before surgery (may be performed on the same day as surgery).

- Physical examination, ECOG performance status and vital signs
- Hematology, hemostatic functions and blood chemistry tests.
    - Hematology: counts of hemoglobin, neutrophils, platelets, leukocytes.
    - Hemostatic functions: PT, PTT, INR.
    - Blood chemistry: AST, ALT, ALP, bilirubin total, direct bilirubin, serum creatinine, albumin-adjusted serum calcium, electrolytes (P-, Mg2+, Na+, K+), albumin, uric acid, random blood sugar.
    - Serum CTX levels

6.4 Surgery visit

- Biological samples:

- Tumor samples: 1-2 frozen and 1-2 FFPE cores. These samples are to be taken from the residual surgical specimen.
- A sample of normal tissue of the breast will also be collected (1 core frozen, 1 core FFPE).
- Blood samples: 10 ml of blood, serum and plasma for future biomarker evaluation.

All AEs whatever their relation to the study drug including SAEs during treatment until 1 month after the last dose must be documented according to NCI-CTCAE v 4.0. Thereafter, only the project specific AEs should be reported until 3 months after the last dose.

Concomitant medications and procedures performed must be documented until 1 month after the last dose.

6.5 After surgery

All procedures related to tumor assessment, patient treatment and follow-up are at the discretion of the investigator. The study will not collect any further information regarding the patient following surgery except for safety data specified in the following sections.

Schedule of Assessments – Screening and Treatment Period

	Screening	Day 8 (7-12)	pre-surgery (day 9-Surgery)	Surgery (day 10-21)
Informed consent (a)	x			
Eligibility criteria	x			
Menopausal status (b)	x	x		
Contraception	x			
Pregnancy test (c)	x			
Demographic, medical history (d)	x			
Physical examination (e)	x	x	x	
ECOG Performance status	x	x	x	
Laboratory analysis (f)	x	x	x	
Radiological examinations (g)	x			
Tumor and normal breast tissue biopsy (h)	x			x
Blood samples (i)	x		x	
Pathological characteristics of primary breast cancer				x
Adverse events (j)	x (if applicable)	Continuously		
Serious adverse events (j)	x (if applicable)	Continuously		
concomitant treatments and procedures (k)	x	Continuously		

a. Signed informed consent must be obtained before any study specific screening assessments are performed.

b. Menopausal status will be determined according to the date of LMP and the biochemical evidence of menopausal status (FSH, LH and estradiol tests). The phase of the menstrual cycle will also be determined at Day 8.

c. For all women of childbearing potential: a serum β-human chorionic gonadotropin (HCG) or a urine pregnancy test must be performed within 7 days prior to the first dose administration.

d. Cancer medical history and any other relevant medical history.

e. Physical examination and vital signs including height (at baseline only), weight, blood pressure, heart rate, respiratory rate and body temperature.

f. Hematology and hemostatic function, blood chemistry (AST, ALT, ALP, bilirubin total, direct bilirubin, creatinine, albumin-adjusted serum calcium, Magnesium, Phosphorous, Sodium, Potassium, Albumin, Uric acid and Random blood sugar), Serum CTX level, Serology at baseline only (HBV, HCV, HIV). At day 8, only albumin-adjusted serum calcium is measured.

g. At least a breast radiological examination is mandatory (mammography, MRI or ultrasound).

h. Tumor and normal tissue biopsy at baseline (tumor: minimum 1-2 core FFPE and 1-2 cores snap frozen; normal 1 frozen, 1 FFPE) and at surgery (tumor 2 cores FFPE and 2 cores snap frozen; normal 1 frozen, 1 FFPE).

i. Blood samples: whole blood, serum and plasma for biomarker evaluation.

j. AEs and SAEs will be collected from the start of study screening procedures. All non-serious AEs occurring prior to study day 1 (administration of study drug) will be reported in the medical history, unless AE reporting is deemed more appropriate. AEs are to be monitored continuously during study treatment up to 1 month after the last administration of study drug. Thereafter only project specific AEs continue to be collected up to 3 months after the last administration of study drug.

k. Concomitant medication and procedures will be recorded from screening up to 1 month after the last administration of study drug.

6.6 Safety

Measurements used to evaluate safety will include vital signs, clinical laboratory tests (hematology, hepatic, renal functions and assessment of albumin-adjusted serum calcium). AEs will be assessed throughout the study from the first administration of the study drug and up to 1 month after the last administration of the study drug. Project specific AEs should also be recorded up to 3 months after last administration of the study drug -see section 8.1.2. If a patient suffers from a grade 3-4 AE, treatment should be stopped.

6.6.1 **Pregnancy**

Baseline serum or urine pregnancy test is mandatory for all patients with childbearing potential within 7 days prior to administering the 1st dose of the drug. This will only be repeated if clinically indicated or as required by the treating physician. All patients should be advised to use active local contraception throughout the study and up to 7 months following the last administered dose.

6.6.2 **Hypocalcaemia**

Serial assessments of serum calcium levels will be performed at baseline, performed within two days before the second administration of study drug and prior to surgery (if more than 7 days after the last testing). All patients are required to take supplementary calcium and vitamin-D during the treatment period to avoid such AE. In case of grade 3 or 4 hypocalcaemia, administration of intravenous calcium is allowed. The second dose of the drug should be canceled and surgery should be delayed until levels are in the normal range.

6.6.3 Osteonecrosis of the Jaw (ONJ)

All patients are encouraged to have a dental checkup prior to enrollment. Dental interventions are strongly recommended against during and up to one month following the second and last injected dose. Invasive dental procedures should also be avoided during the trial duration, but non-invasive procedures are allowed.

7. STUDY TREATMENT

7.1 Investigational product: Denosumab

7.1.1 Description

Denosumab is a human IgG2 monoclonal antibody with high affinity and specificity for RANKL. Denosumab has an approximate molecular weight of 147 kDa and is produced in genetically engineered mammalian (Chinese hamster ovary) cells.

7.1.2 Dosage form and administration

Denosumab is provided as a sterile, colorless to slightly yellow, practically free from particles, preservative-free solution intended for subcutaneous injection. The vial contains 70 mg/mL denosumab, 18 mM sodium acetate and 4.6% (w/v) sorbitol, at a pH of 5.2, filled to a target deliverable volume of 1.7 mL. A 30-gauge needle should not be used, as this may affect the quality of the product.

7.1.3 Supportive care guidelines

No pre-injection supportive care measures are required. All patients must be willing to use supplementary oral calcium ≥ 500mg/day and vitamin D ≥ 400 IU/day up to the day of surgery.

7.1.4 Handling and storage

Denosumab should be stored protected from light and according to the storage and expiration information (where required) provided on the label that is affixed to the package containing the investigational product.

7.2 DRUG INTERACTIONS

No formal drug-drug interaction studies have been conducted with denosumab. In clinical trials, denosumab has been administered in combination with standard anti-cancer treatment and in subjects previously receiving bisphosphonates. The pharmacokinetics and pharmacodynamics of denosumab were not altered by concomitant chemotherapy and/or hormone therapy or by previous intravenous bisphosphonate exposure. The collection of concomitant treatments and procedures will occur up to 1 month after the last administration of the drug.

7.3 SUBJECT COMPLETION AND WITHDRAWAL

The patient may voluntarily discontinue participation in the study at any time. The investigator may also decide to discontinue the patient from participating in the study. The primary reason for study discontinuation should be clearly documented in the study case report form. The subject is considered as off-trial 3 months after the last dose of study drug. AEs will be assessed up to one month after the last administration of the drug. Project specific AEs should also be recorded up to 3 months after the last administration of the study drug -see section 8.1.2.

7.3.1 **Treatment completion**

Investigational treatment(s) will be completed after the second dose prior to surgery or discontinuation for other reasons (e.g., subject refuses further treatment). Once the subject discontinues investigational product, the subject will not be allowed to be retreated.

7.3.2 **Subject Completion**

A patient who has received at least one dose of the study drug will have completed the study in any of the following instances: 3 months after the last dose of study drug, death, lost-to follow up or withdrawal of study consent.

7.3.3 Subject withdrawal

A patient may withdraw from the study or study specific procedures at any time during the entire duration of the study for any reason and without prejudicing future medical treatment.

Whilst a patient can withdraw without needing to give a reason, as soon as a patient has triggered a withdrawal, the investigator has the responsibility to establish that the patient's decision is an informed choice and to ascertain to what extent the patient might be willing to continue limited participation in the trial, e.g., being willing to continue being contacted or seen with the view to providing follow-up information. The outcome of the discussion should be documented in both the patient's medical records and the CRF.

It is therefore important to clarify that patient's withdrawal is defined within two different scenarios that have a different impact on the study analysis and data collection:

• Withdrawal from study treatment: the decision to withdraw from treatment can be taken by the patient or by the investigator. Patients must be kept on study and followed up according to the protocol schedule of assessments until study completion. The reason for treatment discontinuation must be recorded on the CRF. If the patient decides to withdraw from treatment because of an AE, the reason for treatment discontinuation should be reported as AE even in the case the investigator does not consider it as qualifying for treatment withdrawal as per protocol.

Investigators may withdraw patients from study treatment in the event of intercurrent illness, AEs, treatment failure, protocol violation, administrative reasons or for other reasons.

• Withdrawal from the entire study: should a patient decide to withdraw from the study, all efforts will be made to complete and report the observations as thoroughly as possible. No further data will be collected after the date of withdrawal from study.

The Investigator should contact the patient or a responsible relative by telephone or through a personal visit to establish if the patient agrees that the samples already obtained can continue to be used as part of this study. If the patient refuses, the samples must be destroyed. If there is no objection, all the samples collected will be used for the purposes of this study. In case of death or lost to follow-up, where there are no clear wishes to destroy the samples, the samples will also be used for the study.

In the case of patients who does not show up for scheduled visits, several attempts should be made by the site to contact these patients for follow up information. If any of the trial patients are lost to follow up, contact will initially be attempted through the trial research nurse and the lead investigator at each center. Where these attempts are unsuccessful, the patient's general practitioner will be contacted and asked to contact the patient or her/his family and provide follow-up information to the recruiting center.

It is only after sufficient attempts at contact have been unsuccessful, that a patient may be declared "Lost to follow-up".

An excessive rate of withdrawals and loss to follow-up can render the study uninterpretable; therefore, unnecessary patient attrition should be avoided.

7.3.4 Treatment after the end of the study

The end of study is three months after the last dose of drug or if the subject is withdrawn from the study. Once the subject is withdrawn from investigational product, the subject will be treated as determined by the attending physician.

Patients are included in the study as long as they receive at least one dose of the investigational agent.

7.3.5 Investigational Product Accountability

The Investigator or other appropriate delegated individual should maintain records of the inventory at the site, the use for each subject in addition to delivery, storage and destruction of the study drug. Investigators should maintain records that adequately document that subjects were provided the doses specified in the protocol and reconcile all investigational product(s) received from the sponsor.

The Investigator should ensure that the investigation product is used only in accordance with the protocol. The Investigational product should be stored as specified by the Sponsor and in accordance with applicable regulatory requirements. The Investigator should ensure that each subject is adequately informed about the correct use of the Investigational product and check periodically that each subject is following the instructions correctly.

8. SAFETY

For all AEs, SAES and project specific AES, the following details must be assessed and recorded on the AE pages of the Case Report Form: start date, intensity, relationship to study drug, action taken, stop date and outcome of the event.

8.1 Adverse events (AE) and serious adverse events (SAE)

An AE is defined as any untoward medical occurrence in a patient or clinical investigation subject to whom a pharmaceutical product has been administered, and which does not necessarily have to have a causal relationship with this treatment. An AE can therefore be any unfavorable and unintended sign, symptom, or disease temporally associated with the use of a medicinal product, whether or not considered related to the medicinal product. Pre-existing conditions, which worsen during the study, are to be reported as AEs.

AEs should be reported regardless of causality in all patients for until one month after the last dose of investigational therapy.

8.1.1 **Definition of a Serious Adverse Event (SAE)**

A SAE is any untoward medical occurrence that at any dose:

- Results in death;
- Is life-threatening (i.e. the patient was at immediate risk of death at the time the event was observed);
- Requires new hospitalization or prolongation of hospitalization;
- Results in persistent or significant disability/incapacity;
- Is a congenital anomaly/birth defect;
- Is medically important (i.e. important AEs that are not immediately life threatening or do not result in death or hospitalization but may jeopardize the patient or may require intervention to prevent one of the other outcomes listed above).

The term "life-threatening" refers to an event in which the patient was at an immediate risk of death at the time of the event; it does not refer to an event which could hypothetically have caused death had it become more severe. Medical and scientific judgment should be exercised in deciding whether expedited reporting to the sponsor is appropriate in other situations, such as important medical events that may not be immediately life-threatening or result in death or hospitalization but may jeopardize the patient or may require intervention to prevent one of the outcomes listed in the definitions above.

The term "severe" is a measure of intensity, thus a severe AE is not necessarily serious. For example, nausea of several hours' duration may be rated as severe, but may not be clinically serious. Hospitalization for the performing of protocol-required procedures or administration of study treatment is not classified as an SAE.

8.1.2 **Project specific AEs**

The starting period for collecting AEs is from signing the ICF. The study ends with the surgical procedure. AEs of any causality must be reported up to 1 month after the

last administration of the drug. However, it is a special follow-up requirement of this study to report any wound healing infections, AEs during surgery or post-anesthesia period or abnormal events occurring up to 3 months after the last dose of investigational therapy.

Events of interest include:

- Osteonecrosis of the jaw (ONJ)
- Hypocalcaemia
- Infections, particularly of related to the surgical procedure
- Prolonged hospital stay post-surgical procedure
- AEs during surgery or post-anesthesia
- Readmission up to one month from the last administration of the drug

8.1.3 Clinical and laboratory abnormalities as AEs and SAEs

All laboratory findings performed will be reported in the CRF. The investigator is responsible for reviewing laboratory test results and determining whether an abnormal value represents a clinically relevant change from values before the initiation of study drug. In general, abnormal laboratory findings without clinical significance (based on the investigator's judgment) should not be recorded as AEs; however, laboratory value changes requiring therapy or adjustment in prior therapy are considered AEs. Abnormal laboratory findings (e.g., Hematology, hemostatic function, blood chemistry, and serology) or other abnormal assessments (e.g., vital signs) that are judged by the investigator as clinically significant will be recorded as AEs or SAEs. Clinically significant abnormal laboratory findings or other abnormal assessments that are detected during the study or are present at baseline and significantly worsen following the start of the study will be reported as AEs or SAEs. However, clinically significant abnormal laboratory findings or other abnormal assessments that are associated with the disease being studied, or are present or detected at the start of the study and do not worsen, will not be considered as AEs or SAEs. The investigator will exercise his or her medical and scientific judgment in deciding whether an abnormal laboratory finding or other abnormal assessment is clinically significant.

All grade 4 laboratory abnormalities will be reported as SAEs.

8.1.4 Causality of AEs

The relationship of an AE to the treatment should be assessed by the investigator.

8.1.5 Grading of AEs

The National Cancer Institute Common Terminology Criteria for AEs (NCI-CTCAE Version 4.0 Appendix 2) will be used for grading of AEs. For AEs for which the CTCAE does not provide a grading scale, the severity will be graded according to the following four-point scale and reported in detail as indicated in the CRF:

- 1= “Mild”: discomfort noticed but no disruption of normal daily activity;
- 2= “Moderate”: discomfort sufficient to reduce or affect daily activity;
- 3= “Severe”: inability to work or perform normal daily activity;
- 4= “Life-Threatening”: represents an immediate threat to life.
- 5= “Death”: results in death.

8.1.6 Reporting Procedures for All AEs

The investigator is responsible for ensuring that all AEs observed by the investigator or reported by subjects are properly captured in the subjects’ medical records.

AEs have to be reported from the day of signed informed consent until one month after the last administration of study drug. Protocol specific AEs should be reported until 3 months after the last administration of study drug.

The following adverse event attributes must be assigned by the investigator: AE diagnosis or syndrome(s) (if known, signs or symptoms if not known); dates of onset and resolution; severity; assessment of relatedness to study treatment; and action taken.

Medically significant AEs considered related to the study or the investigational product by the investigator or the sponsor will be followed until resolved or considered stable or until database lock. It will be left to the investigator’s clinical judgment to determine whether an AE is related and of sufficient severity to require the subject’s removal from treatment or from the study. A subject may also

voluntarily withdraw from treatment due to what she perceives as an intolerable AE. If either of these situations arises, the subject should be strongly encouraged to undergo an end-of-study assessment and be under medical supervision until symptoms cease or the condition becomes stable.

8.1.7 Serious Adverse Events Reporting Procedures

The investigator should notify the Sponsor of all SAEs occurring at the site(s) in accordance with local procedures, statutes and the European Clinical Trial Directive (where applicable). The Sponsor will medically review all SAEs.

The Sponsor will ensure the notification of the appropriate Ethics Committees, Competent Authorities and participating Investigators of all SAEs occurring at the site(s) in accordance with local legal requirements, statutes and the European Clinical Trial Directive.

SAEs have to be reported from the day of the signed informed consent until one month after the last administration of study drug. Protocol specific AEs should be reported until 3 months after the last administration of study drug. All the AEs will be followed until resolution for a maximum of 3 months following the last dose of denosumab.

The study sponsor is responsible for providing all suspected serious adverse drug reactions (SADRs) occurring in subjects exposed to the Amgen product to the Amgen local safety officer within one month of the event. It is possible that Amgen may request follow-up information from the sponsor.

All suspected unexpected serious adverse reactions related or possibly related to denosumab (SUSARs) and their follow-up reports must be reported to Amgen at the same time as submission to the regulatory agency, IRB or IEC. A copy of any safety report involving an Amgen drug submitted to the regulatory agency, IRB or IEC, should be faxed to Amgen, within 24 hours of such submission. The sponsor is responsible to ensure that the latest investigator's brochure is used as the source document for determining the expectedness of an SAE.

8.2 Reporting of pregnancy

The Sponsor must report pregnancy of a subject participating in the clinical trial to Amgen within 15 days of identification. Follow up case reports have to be provided as new information becomes available. While pregnancy itself is not considered to be an AE or SAE, any pregnancy complication or elective termination of a pregnancy for medical reasons will be recorded as an AE or SAE. A spontaneous abortion is always considered to be an SAE and will be reported as such.

8.3 Death

A death occurring during treatment with the investigational drug or within three months after the last administration of the drug, whether considered to be treatment-related or not, must be reported. All deaths thought to be related to study drug at any time should be reported and the cause of death must be reported as a SAE regardless of the time elapsed since the last dose of investigational product until database lock. Deaths related to progression of the underlying disease during the course of the study will not be reported as a SAE, but should be reported on the appropriate CRF page (unless the patient has withdrawn consent). Deaths from other causes should be reported on the appropriate CRF page.

9. DATA ANALYSIS AND STATISTICAL CONSIDERATIONS

9.1 Analysis sets

9.1.1 Per-protocol Set

The Per-protocol analysis set that will be used for all objectives concerning biomarkers consists of all patients that are registered for study participation and have fulfilled the following criteria:

- Patient meets eligibility criteria
- Patient has received the two specified doses of the study drug

- Adequacy for molecular assessment of the tissue specimens collected before and after the study treatment initiation. The adequacy will be evaluated by the central laboratory according to the following criteria:
  i. Sufficient tissue to perform Ki67 IHC
  ii. 1.0 µg of total RNA available of appropriate quality
- Blood samples are collected as specified in Section 9 and detailed in the SPAM are sent to the central laboratory
- Serum samples for CTX levels are collected as specified in Section 9 and detailed in the SPAM are sent to the central laboratory

9.1.2 Safety Set

The Safety set will include all subjects who received at least one dose of study medication.

9.2 Primary endpoint and sample size determination

39 pre-menopausal women will be enrolled to ensure at least 34-paired evaluable samples.

The sample size is calculated in order to estimate the geometric mean decrease in percentage stained Ki67 values assessed by IHC in the tumor with a certain precision (i.e. width of a 95% confidence interval around the mean decrease in logged Ki67 values from baseline to prior to surgery, back-transformed to the original scale).

We performed an analysis of the data of a 14-days letrozole alone window study in order to estimate the expected variability in the change in logged Ki67 IHC in this window study (Bedard et al., 2011; Miller et al., 2009). In that study, the geometric mean decrease in Ki67 values after 14 days of letrozole alone was given by 29% (95% CI 22%-38%) among 51 patients diagnosed with estrogen receptor positive breast cancer. The observed standard deviation of the change in logged Ki67 IHC after 14 days of letrozole alone treatment was 0.98. In order to estimate the mean decrease in Ki67 IHC by denosumab with a 95% confidence interval of a width of 0.66, a total of 34 evaluable patients are needed.

9.2.1 Accrual rate and study duration

Participation of three to four institutions would allow a 1-year recruitment period.

9.3 Secondary endpoints

For all secondary endpoints 95% confidence intervals will be calculated. Normal distributions of logged values will be visually inspected. Pre- and post-treatment values will be compared using a paired t-test or a Wilcoxon signed rank test if there was departure from normal distribution hypothesis.

9.3.1 Key secondary endpoints

- Absolute Ki67 responders after a short course of denosumab treatment, defined as <2.7% Ki67 IHC staining in the post treatment tumor biopsy.
- Decrease in serum C-terminal telepeptide (CTX) levels measured by ELISA will be assessed by change in levels from baseline to prior to surgery. Percentage change from baseline will be reported as the median (IQR) with statistical significance to be calculated using the Sign test.
- Change in IHC expression of RANK/RANKL changes in signaling from baseline to prior to surgery will be assessed using both percentage stained and intensity of staining [Histo-score (Rojo et al., 2007)] in the tumor. Change in gene expression of RANK/RANKL single genes and a gene module will also be evaluated.

- Change in tumor proliferation rates at the gene expression level will be assessed using single genes (AURKA, Ki67) and gene modules using the Genomic Grade Index (GGI) (Sotiriou et al., 2006).
- Change in tumor apoptosis rates will be evaluated with the use of TUNEL and caspase-3 staining using IHC.

9.3.2 Other secondary endpoints

- Change in genes representing immature mammary stem cell populations in the tumor

- Gene modules corresponding to immature mammary epithelial cell populations (MaSC and luminal progenitors) as described in Lim et al., 2009.
- Change in IHC expression of stem cell markers such as ALDH1.

- Change in estrogen pathway-related genes in the tumor
  - Using estrogen receptor (ER) IHC
  - single genes (ESR1, PgR, BCL2) gene expression
  - and estrogen-related gene sets using gene expression (SET index) (Symmans et al., 2010)
- Change in tumor infiltrating lymphocytes (TIL) in the tumor
  - TILs will be quantitated using full face H&E sections (Denkert et al., 2010)
- Change in expression of T regulatory cells in the tumor
  - Specific immune genes related to T regulatory function to be defined in the statistical analysis plan (for example: CD4, FOXP3,) using both gene expression and IHC
  - Immune-related gene sets using gene expression data (STAT1 and IRM modules) (Desmedt et al., 2008; Teschendorff et al., 2007).
- Safety data will be evaluated using the National Cancer Institute Common Terminology Criteria for Adverse Events (NCI-CTCAE v 4.0) particularly:
  - Osteonecrosis of the jaw
  - Hypocalcaemia
  - Infections
  - Prolonged hospital stay post-surgical procedure
  - AEs during surgery or post-anesthesia
  - Readmission up to one month from the last administration of the drug
- All the above comparisons will be made in surrounding normal tissue pairs
- All the above endpoints in surrounding normal tissue will be compared with that occurring in the tumor.

The Affymetrix gene expression data will be analyzed in using R statistical software. The data will be normalized using the frozen RMA (McCall et al., 2010) and log2 transformed. The mean absolute decrease from baseline to prior to surgery in log2 expression will be calculated and a confidence interval estimated.

9.4 Subgroup analysis and effect of baseline factors

To determine whether the results for the primary factors are consistent across various subgroups, subgroup analysis may be performed within each category of the following classification variables:

- ER status: positive (>1%) vs. negative
-PgR status positive (>1%) vs. negative
- Her2 status: positive (+3 by IHC or FISH-positive) vs. negative
- Phase of menstrual cycle: Follicular phase (including ovulation) vs. luteal phase

In addition to this, multivariate analyses will be performed adjusting for baseline characteristics: ANCOVA (Analysis of Covariance) models will be applied to all endpoints listed above in order to estimate the effect of treatment on post-treatment values while adjusting for baseline values and important clinico-pathological characteristics. If after visual inspection the residuals from the ANCOVA model do not follow a normal distribution, a non-parametric ANCOVA will be applied.

9.5 Exploratory analysis

Exploratory analyses will also be performed according to groups defined by:

- PgR status (positive vs. negative)
- RANKL status (IHC positive vs. negative) in normal breast tissue
- RANKL status (IHC positive vs. negative) in infiltrating cells or stroma
- RANKL status (IHC positive vs. negative) in tumor tissue
- RANK status (IHC positive vs. negative) in normal tissue
- RANK status (IHC positive vs. negative) in tumor tissue

- Above changes according to phase in the menstrual cycle (follicular vs. luteal)

An exploratory gene set enrichment analysis (GSEA) will be applied to discover gene sets from pathways whose genes show concordant differences prior to surgery from baseline (paired GSEA analysis).

Further details will be included in the final statistical analysis plan.

10. TUMOR TISSUE HANDLING

10.1.1 Biological samples

Biological samples will be collected at baseline and at the definitive surgical procedure.

For each time point sample collection will be done as follows:

1) Frozen tumor tissue (1-2 core snap frozen embedded in OCT)
2) Formalin-fixed paraffin-embedded tumor tissue (1-2 cores FFPE)
3) Normal breast tissue (at least 1cm from tumor- different quadrant, contralateral breast are acceptable-1 core snap frozen embedded in OCT, 1 core FFPE)
4) Whole blood
5) plasma
6) serum

Biological tissue, whole blood, plasma and serum will be collected for each patient and will be submitted for translational studies. Plasma, serum and whole blood samples will be stored for future assessment of circulating tumor markers.

Normal breast tissue will be requested to be collected at baseline and from surgery in case comparisons need to be made with normal breast tissue.

10.1.2 Markers to be assessed

The following markers will be evaluated in the tumor specimen for their changes from baseline to prior to surgery:

- Proliferation markers
  - Ki67 as a single marker using gene expression and IHC as recommended by Dowsett and colleagues (Dowsett et al., 2011)
  - Proliferation gene signatures using gene expression such as the GGI (Sotiriou et al., 2006)
- RANK/RANKL signaling using gene expression of individual genes, a gene module (developed using a method described previously in Desmedt et al, 2008, or if new published data emerges) and IHC.
- Apoptosis using the terminal deoxynucleotidyl transferase-mediated nick-end labeling-assay (TUNEL) with the ApopTag Plus Peroxidase In Situ Apoptosis Detection Kit (Chemicon International, Temecula, CA) and caspase-3 immunohistochemical staining (Abcam ab2302)
- Modulation in immature mammary epithelial cell populations using genes and gene sets at the RNA level and using IHC for specific stem cell markers (i.e. ALDH-1) (Lim et al., 2009)
- Estrogen pathway-related genes
  - Using single genes (ESR1, PgR) with both gene expression and IHC
  - Estrogen-related gene sets using gene expression (Symmans et al., 2010)
- Change in tumor infiltrating lymphocytes (TILs)
  - Total TILs will be quantified using full face H&E sections for both intra-tumoral and stromal lymphocyte percentage infiltration (Denkert et al., 2010)
  - specific T regulatory immune-related genes to be defined in the statistical analysis plan using both gene expression and IHC
  - Immune-related gene sets using gene expression data (STAT1, IRM, and others) (Desmedt et al., 2008; Teschendorff et al., 2007)

The above will also be evaluated in the paired normal tissue biopsies as well as a comparison made between relative changes occurring in normal and tumor tissue.

Further details will be included in the final statistical analysis plan.

10.1.3 **Methods for assessment**

An H&E section will be taken from all samples to document the percentage of tumor, stromal and adipose components. The following table will be completed by the pathologist at the Jules Bordet Institute on all specimens for each core biopsy taken. Importantly, the pathologists will have to evaluate the % of tumor nuclei of each component that make up the tumor and not the % of tumor surface.

**Component**	**% of nuclei for each component (must add to 100%)**
TUMOR- INVASIVE	
DUCTAL CARCINOMA IN SITU (DCIS)	
LYMPH TISSUE	
STROMA	
ADIPOSE	
NORMAL EPITHELIAL	

The following variables will be assessed at baseline and surgery on frozen tissue

Variable	≠ probe sets	Description	Cut-off point for determining sensitivity
RIN		RNA Integrity Number	7
3'5'actin	2	House Keeper RNA 3'to 5' probe set ratio	NA
3'5'GAPDH	2	House Keeper RNA 3'to 5' probe set ratio	NA
3'5'18Sr RNA	2	House Keeper RNA 3'to 5' probe set ratio	NA
RANKL	1	RANK-ligand	Median
ALDH1	1	Aldehyde dehydrogenase 1	Median
ESR1	1	Estrogen receptor mRNA (ERα)	As published (Karn et al., 2010)
ERBB2	1	HER2 gene (mRNA)	As published (Karn et al., 2010)
Ki67	1	Proliferation gene (mRNA)	Median
AURKA	1	Proliferation gene (mRNA)	Median
CD4	1	Immune-related gene (mRNA)	Median
FOXP3	1	Immune-related gene (mRNA)	Median
CD45	1	Immune-related gene (mRNA)	Median
CXCL13	1	Immune-related gene (mRNA)	Median
GGI index	96	Genomic grade index	As published (Sotiriou et al., 2006)
Luminal progenitor get set	Varied	Developed by (Lim et al., 2009)	NA
Mammary stem cell gene set	Varied	Developed by (Lim et al., 2009)	NA
IRM	Varied	Immune related module developed by (Teschendorff et al., 2007)	NA
STAT1	Varied	(Desmedt et al., 2008)	NA

The composition of a RANK/RANKL signaling module is to be determined

NA: to be recorded or not assessable, since no formal cut-off has been validated, the variable will only be analyzed in its continuous form

The following variables will be assessed at baseline and surgery on FFPE

Variable	IHC antibody	Description	Scoring method
RANK/RANKL	AMGEN	To be performed by AMGEN	
ER		As CLIA certified by lab in IJB	Percentage stained, intensity, Allred score
PgR		As CLIA certified by lab in IJB	Percentage stained, intensity, Allred score
Ki67	DAKO	Proliferation marker	Percentage stained
Caspase-3	Apcam ab2302	Apoptosis marker	Percentage stained
TUNEL	Chemicon	Apoptosis marker	Percentage stained >200 is intense apoptosis
ALDH1	BD biosciences	Stem cell marker	Percentage stained

10.1.4 Ki67 Immunohistochemistry

Immunohistochemistry (antibody DAKO MIB1) will be performed automatically

(Ventana Medical Systems) to assess proliferative activity in paraffin-embedded samples. This staining will be performed within an ISO15189-accredited environment with validated procedures, equipment and documented technical and interpretative competence of all personnel involved (medical and non-medical staff). The percentage of all tumor cells staining with this antibody will be manually determined by an experienced pathologist blinded to the clinical data. Methods will be followed as described in the recent white paper on Ki67 (Dowsett et al., 2011). Ki67 is a commonly performed IHC test in the pathology department of the central lab.

10.1.5 **Apoptosis markers**

Immunohistochemistry (antibody Abcam ab2302) will be performed automatically (Ventana Medical Systems) to assess apoptotic activity in paraffin-embedded samples. This staining will be performed within an ISO15189-accredited environment with validated procedures, equipment and documented technical and interpretative competence of all personnel involved (medical and non-medical staff). The percentage of all tumor cells staining with this antibody will be manually scored since a high correlation has been shown with the percentage of caspase-3 positive apoptotic bodies. The obtained results will be analyzed as a continuous variable since no formal cut-off using this method has already been validated. Implementation of the TUNEL with the ApopTag Plus Peroxidase In Situ Apoptosis Detection Kit (Chemicon International, Temecula, CA) will be foreseen and results will be compared with the caspase-3 data. The level of apoptosis will be evaluated on a 0 to 400 scale. Intense apoptotic activity using this method is defined as a score of more than 200. Absolute number of apoptotic cells will also be counted for each sample and recorded.

10.1.6 **Tumor infiltrating lymphocytes**

Also a H&E slide from FFPE biopsies will be prepared to assess TILs using whole sections as previously described (Denkert et al., 2010). Stromal lymphocytes and intra-tumoral lymphocytes will be recorded as percentage infiltration and compared pre and post therapy.

10.1.7 **Blood samples**

Blood samples for translational studies will be obtained at the same time points as for tumor samples (i.e. at baseline and surgery). Whole blood, serum and plasma will be collected at baseline, prior to treatment and prior to surgery. These will be stored for future research purposes in case an interesting circulating biomarker to RANKL inhibition and prognosis is reported in the near future (i.e. circulating tumor cells, circulating nucleic acid or germline SNP). In the future, host genetic factors may also be discovered that may predict response to denosumab.

10.1.8 **Serum CTX levels**

Serum samples will be obtained at the same time points as for tumor samples, i.e. baseline, and at surgery (or a pre-surgery: 1 day from the second and last dose of denosumab). If local testing cannot be performed, samples should be stored at -70˚C and submitted for central assessment of CTX levels testing (please inform the study data manager).

10.2 Optional Translational Fresh Tissue Sub-study

A sub-study will occur for interested sites, where feasible, where fresh tumor and normal tissue will be collected from the patients participating in the study at the time of surgery for flow cytometry. Procedures for this sub-study will be outlined in the SPAM.

We shall use 10-color flow cytometry to assess cellular subpopulations in homogenates of fresh tumor and normal tissue taken at surgery. Marker expression for stem cell, epithelial cells, stromal cells, RANKL positive and different immune response subpopulations will be quantified in both tissues and blood (the latter for immune markers only). The supernatant from these fresh tissue homogenates will be retained and examined for a reproducible pattern of cytokine/chemokine expression using flow cytometric beads. Markers are to be defined in collaboration with Amgen.

10.3 Tissue samples (please refer to the SPAM)

10.3.1 Sample collection

BASELINE: A minimum of 1-2 frozen and 1-2 FFPE cores using a 14-gauge or smaller diameter spring-loaded biopsy needle will be taken. Two biopsies are preferable and the priority should be at least one frozen and one FFPE and then the next priority should be a frozen sample. Each core should be >1 cm x 1 mm and it is required that at least 1/3 of the cylinder should be infiltrated by the invasive component of the primary tumor. During the biopsy procedure, multiple passes of the needle may be necessary to adequately obtain samples of sufficient quality for molecular analysis. Failure to obtain samples of sufficient quality during the screening phase will render the patient ineligible for the trial.

In some cases, tissue suitable for molecular studies may have been obtained during a routine diagnostic procedure performed prior to study enrollment (in between 10-21 days). In these cases, the patient does not need to be re-biopsied to participate in this study provided the patient has signed consent for her tissue to undergo the molecular tests foreseen in the trial.

A sample of normal tissue (1 core frozen, 1 core FFPE) is also requested. This should be taken at least 1 cm away from the tumor, (defined as at least 1cm away from tumor). This may be taken near the tumor, in another quadrant of the breast or the contralateral breast. If possible, the surgical biopsy should be taken from a similar area.

SURGICAL: At surgery, 1-2 additional frozen and 1-2 additional FFPE cores will be taken. Priorities should be the same as the baseline sample. These samples are to be taken from the residual surgical specimen (i.e. not required for diagnostic purposes).

A specimen of breast tissue (1 core frozen, 1 core FFPE) should also be taken, at least 1cm away from the tumor bed, in a similar location to the baseline biopsy. These

specimens will be obtained and processed as described in detail in the SPAM. Paired samples will be shipped together to the central laboratory (Jules Bordet Institute).

PROCESSING: Once the samples are received at the central laboratory at Jules Bordet Institute, an H&E slide will be obtained from both the frozen and FFPE samples and it will be evaluated by a pathologist according to the criteria described in the SPAM. RNA and DNA will be extracted and their quality and quantity will be evaluated. Gene expression profiling will be obtained using the Affymetrix® U133 Plus 2.0 arrays or using next generation sequencing technologies.

Standardized protocols should be applied consistently in preparing and storing biospecimens to ensure quality and to avoid introducing variables into research studies. Biospecimen resource personnel should record storage conditions along with any deviations from the standard operating procedures (SOP), including information about temperature, thaw/refreeze episodes, and equipment failures.

10.4 Sample transportation

10.4.1 Frozen tissue

When seeking to regulate sample temperature during shipping, the shipping time, distance, climate, season, method of transportation, and regulations as well as the type of samples and their intended use should be considered (Landi and Caporaso, 1997). To maintain proper temperature during shipping, appropriate insulation, gel packs, dry ice, or liquid nitrogen (dry shipper) may be used. For frozen temperatures at -80 °C, dry ice pellets or sheets should be used; dry ice is considered a hazardous substance for shipping purposes. Whenever intending to maintain samples below ambient temperature, enough refrigerant should be included to allow for a 24-hour delay in transport (ISBER, 2008). Temperature-sensitive material should be handled by a courier with resources to replenish the refrigerant in case of a shipping delay (ISBER, 2008). A simple colorimetric or other temperature-measuring device should be included with biospecimen shipments to indicate the minimum and/or maximum temperature within the shipping container.

10.4.2 **FFPE**

Paraffin blocks may be shipped at room temperature in an insulated package via overnight carrier. The use of insulated packages is considered important to minimize the effect of temperature fluctuations and to protect the blocks from temperatures higher than 27 °C. Inclusion of a simple maximum temperature indicator in each package and documentation of the maximum temperature upon receipt are recommended.

10.4.3 **Sample storage**

Samples and material left over after molecular analysis will be stored in the Biobank in the Anatomical Pathology Department of Jules Bordet Institute, Brussels, Belgium. In turn, the investigator must promptly inform the Translational Research Project Manager so that appropriate follow-up can be initiated. The investigator will be informed when the associated specimens are destroyed and should notify the subject that specimen destruction is complete.

10.4.4 **Translational Fresh Tissue Sub-study**

For this sub-study, the tissue must be processed immediately. The fresh tissue is removed in the Anatomical Pathology department and the fragment placed immediately into tubes containing 3ml of X-vivo 20 and returned to the refrigerator (4C) until transport and immediate processing. We will supply information for organizing direct transport of the materials. The subsequent cell purifications and flow cytometric analyses are done immediately (same day). These experiments cannot be done on frozen materials. For further information, please see the SPAM.

11. STUDY CONDUCT AND CONSIDERATIONS

11.1 Data handling and record keeping

11.1.1 **Investigator's file**

The investigator must maintain adequate and accurate records to enable the conduct of the study to be fully documented and the study data to be subsequently

verified. These documents should be classified into two different separate categories: Investigator's study file and patient clinical source documents.

The investigator's study file should be established according to the ICH-GCP E6 and the list of essential documents to be collected and kept up-to-date in this file will be provided to the investigator; Subject clinical source documents would include subject hospital/clinic records; physician's and nurse's notes; appointment book; original laboratory reports; signed informed consent form and consultant letters..

11.1.2 **Case Report Form (CRF)**

The CRF will be either in a paper version or in an electronic version (eCRF). In case of an eCRF, it will be completed electronically with a personal access code. An audit trail will maintain a record of the initial entries and changes made, reasons for change, time and date of entry, and user name of person authorizing entry or change. In case of a paper CRF, all form should be typed or filled out using indelible ink, and must be legible. Errors should be crossed out but not obliterated, the correction inserted, and the change initiated and dated.

For each subject enrolled, a CRF must be completed by the investigator or authorized delegate from the study staff. The Investigator is responsible to make a delegation log mentioning responsible persons to complete and sign the CRF. The investigator should ensure the accuracy, completeness, legibility and timeliness of the data reported in the CRFs and in all required reports. Data reported in the CRF must be derived from source documents and should be consistent with the source documents. Any change or correction to a CRF should be dated, initialed and explained (if necessary). The Investigator will retain a copy of the printed and signed CRF. The Investigator will also retain copies of any data query forms (e.g. lab tests). For all patients the monitor will check the CRFs.

11.1.3 Data collection

BrEAST (Breast European Adjuvant Study Team) Data Centre (located at the Jules Bordet Institute) will perform the data collection, the data management and will hold the clinical study database in agreement with the EU Directive 2001/20/EC and the applicable IJB/BrEAST SOP with the objective of removing errors and inconsistencies in the data which would otherwise impact on the analysis and reporting objectives, or credibility of the Clinical Study Report.

For classification purposes, preferred terms will be assigned by the BrEAST Data Centre to the original terms entered on the CRF, using the most up to date version of MedDRA for AEs and diseases and the INN (international non-proprietary name) drug terms and procedures dictionary for treatments and surgical and medical procedures.

Centralized procedures will be used to confirm patient eligibility, to determine the outcome and to assure the high standard of the reporting of data.

11.1.4 Retention of Documents

Following closure of the study, the investigator or the head of the medical institution (where applicable) must maintain all site study records, except for those required by local regulations to be maintained by someone else, in a safe and secure location. The records must be maintained to allow easy and timely retrieval, when needed (e.g., audit or inspection), and, whenever feasible, to allow any subsequent review of data in conjunction with assessment of the facility, supporting systems, and staff. Where permitted by local laws/regulations or institutional policy, some or all of these records can be maintained in a format other than hard copy (e.g., microfiche, scanned, electronic); however, caution needs to be exercised before such action is taken. The investigator must assure that all reproductions are legible and are a true and accurate copy of the original and meet accessibility and retrieval standards, including re-generating a hard copy, if required. Furthermore, the investigator must ensure there is an acceptable back-up of these reproductions and that an acceptable quality control process exists for making these reproductions.

IJB/BrEAST will inform the investigator of the time period for retaining these records to comply with all applicable regulatory requirements. The minimum retention time will meet the strictest standard applicable to that site for the study, as dictated by any institutional requirements or local laws or regulations; otherwise, the retention period will default to 15 years after the completion of the study and/or 2 years after approval by relevant Health Authorities, whichever is longer.

Should the Investigator wish to assign the study records to another party or move them to another location, the Sponsor must be notified in advance. If the Investigator cannot guarantee this archiving requirement at the investigational site for any or all of the documents, special arrangements must be made between the Investigator and the Sponsor to store these in a sealed container(s) outside of the site so that they can be returned sealed to the Investigator, in case of a regulatory audit. Where source documents are required for the continued care of the patient, appropriate copies should be made before storing outside of the site.

11.2 Sponsor's Responsibilities

The IJB will act as the Sponsor for this study. As such:

- It agrees to provide the investigator with sufficient material and support to permit the investigator to conduct the study according to the agreed protocol.
- It reserves the right to request the withdrawal of a patient due to protocol violations, administrative or other reasons.
- It reserves the right to terminate the study prematurely due to persistent protocol violations, administrative or other reasons. Should this be necessary, the procedures will be arranged after review and after consultation by both parties to ensure protection of the patients' interests.
- Upon completion or premature discontinuation of the study, IJB will promptly inform all other investigators or the head of the medical institution (where applicable), and/or institutions conducting the study if the study is suspended or terminated. If required by applicable regulations, the investigator or the

head of the medical institution (where applicable) must inform the IRB/EC promptly and provide the reason for the suspension or termination.

- IJB, as the Sponsor of the Study, has taken insurance in order to cover the activities of the Study.

IJB shall act as the Sponsor of the Study in Belgium according to the Belgian law of May 7, 2004 in agreement with the EU Directive 2001/20/EC.

11.3 Investigator's Responsibilities

The investigator agrees to conduct the study in accordance with the procedures and requirements laid out in this protocol. In particular, the investigator agrees to conduct the study in accordance with strict ethical principles.

It is the responsibility of the investigator to complete the CRFs for each patient in the study, and when a patient completes the study, the investigator must sign all CRFs. The investigator must comment on any missing, unused or spurious data on the appropriate CRF.

Copies of all study-related documents shall be kept by the investigator for the maximum period of time permitted by the hospital, institution or private practice. All documentation and materials provided by IJB for this study are to be retained in a secure place and treated as confidential material.

The investigator has the right to request termination of the agreement for administrative or other reasons. Should this be necessary and agreed upon, the procedures will be arranged after review and after consultation by both parties, to ensure protection of the patients' interests.

By signing this document the investigator indicates that he/she has read the protocol, fully understands the requirements and agrees to abide by all protocol requirements.

The investigator must notify BrEAST Data Centre any changes in the archival arrangements, including, but not limited to, the following: archival at an off-site facility, transfer of ownership of the records in the event the investigator leaves the site.

11.4 Ethical aspects

11.4.1 Local Regulations/Declaration of Helsinki

The Investigator will ensure that this study is conducted in full conformance with the principles of the "Declaration of Helsinki" or with the laws and regulations of the country in which the research is conducted, whichever affords the greater protection to the individual. The study must fully adhere to the principles outlined in "Guideline for Good Clinical Practice" ICH-E6 Tripartite Guideline (January 1997) or with local law if it affords greater protection to the patient and in compliance with the EU Clinical Trial Directive (2001/20/EC).

11.4.2 Central and Local ethics committees

This protocol and any accompanying material provided to the patient (such as patient information sheets or descriptions of the study used to obtain informed consent), as well as any advertising or compensation given to the patient, will be submitted by the Investigator to an Central Ethics Committees, as per national legislation. Approval from the committee must be obtained before starting the study and should be documented in a letter to the Investigator specifying the date on which the committee met and granted the approval.

Each center must undergo assessment by the relevant Local Ethical Committee.
Any modifications made to the protocol after receipt of the Central Ethical Committees approval must also be submitted by the Investigator to the Committee in accordance with local procedures and regulatory requirements.

11.4.3 Informed consent process

11.4.3.1 General

Informed consent is a process initiated prior to an individual agreeing to participate in a trial and continues throughout the individual's participation. In obtaining and documenting informed consent, the investigator should comply with applicable regulatory requirements and should adhere to good clinical practice (GCP) guidelines and to the ethical principles that have their origin in the Declaration of Helsinki.

Potential participants will generally be identified during consultation and cases discussed during the Multi-Disciplinary Team (MTD) meetings. The investigator will provide potential participants with written information about the trial; including contact details of the research team should they require further information, and arrange for an outpatient clinic appointment in the usual way.

Patient Information and Consent forms (PIC), approved by the local ethical committee, will be issued by the investigator. The PIC will describe in detail the trial procedures, the interventions/products, and the potential risks/benefits. All patients will receive the appropriate version of the written information and be asked to read and review it. The PIC will emphasize that participation in the trial is voluntary and that the patient may, without being subject to any resulting detriment, withdraw from the trial at any time by revoking the informed consent. The rights and welfare of the patients will be protected by emphasizing to them that the quality of medical care will not be adversely affected if they decline to participate in this study. All patients will be given the opportunity to ask questions and will be given sufficient time to consider trial entry before consenting.

The consent form will request permission for the patient's General Practitioner to be informed of their involvement in the trial and also permission for personnel involved in the research or from regulatory authorities to have access to the individual's medical records.

For patients not qualified to give or incapable of giving legal consent, written consent must be obtained from the legal representative. In the case where both the patient and his or her legal representative are unable to read, an impartial witness should be present during the entire informed consent discussion.

11.4.3.2 Prior to Trial Registration

The consent process at this stage will be carried out by a member of the research team identified in the trial signature and delegation log. Discussion of objectives and potential inconveniences of the maximum 3 weeks intervention period, and the

conditions under which it is to be conducted, are to be provided to patients by staff with appropriate experience. Patients consent will be obtained prior to any study related procedure being carried out.

Either the delegated staff member or the PI taking consent and the patient must personally sign and date the form.

The original ICF will be filed in the investigator site file. A copy of the signed and dated consent forms will be given to the patient.

If new safety information results in significant changes in the risk/benefit assessment, the consent form should be reviewed and updated if necessary. All patients, including those already being treated, should be informed of the new information, given a copy of the revised form, and give their consent to continue in the study.

11.5 Quality Control and Quality Assurance

11.5.1 Quality control: on-site monitoring visits

In accordance with applicable regulations, GCP and any contracts governing the study, a representative from IJB will contact the site prior to the start of the study to review with the site and staff the protocol, study requirements, and their responsibilities to satisfy regulatory, ethical, and IJB requirements. When reviewing data collection procedures, the discussion will also include identification, agreement and documentation of data items for which the CRF will serve as the source document.

BrEAST will monitor the study consistent with the demands of the study and site activity to verify that the:

- Data are authentic, accurate, and complete;
- Study is conducted in accordance with the currently approved protocol and any other study contracts, GCP, and all applicable regulatory requirements.

Throughout the study, the joint clinical science team will review data according to the Data management SOPs of the BrEAST data centre. The investigator and the head of the medical institution (where applicable) agrees to allow the monitor direct access to all relevant documents.

11.5.2 Quality assurance

Internal: The overall procedures for quality assurance of clinical study data are described in the Sponsors SOPs and the SOPs of the BrEAST Data Centre who will be performing data collection and will hold the clinical study database. To ensure compliance with the protocol, CRF completion guidelines, SOPs, GCP and all applicable regulatory requirements, IJB may conduct a quality assurance audit. External: Regulatory agencies may also conduct a regulatory inspection of this study. Such audits/inspections can occur at any time during or after completion of the study. If an audit or inspection occurs, the investigator and institution agree to allow the auditor/inspector direct access to all relevant documents and to allocate his/her time and the time of his/her staff to the auditor/inspector to discuss findings and any relevant issues.

11.6 Publication policy

Publications and oral presentations of any results from the study shall be in accordance with accepted scientific practice, academic standards and customs and in accordance with the specific policy developed for the study. This policy shall be approved by the Principal Investigators and made available to all investigators/sites and groups participating in the study.

11.7 Finance and Insurance

For details refer to site study agreement.

11.8 Confidentiality of trial documents and subject records

The investigator must assure that subjects' anonymity will be maintained and that their identities are protected from unauthorized parties. On CRFs or other

documents submitted to data management, subjects should not be identified by their names, but by an identification code. The investigator should keep a subject enrollment log showing codes and names. The investigator should maintain documents not for submission to data management, e.g., subjects' written consent forms, in strict confidence.

12. REFERENCES

Aapro, M., Abrahamsson, P.A., Body, J.J., Coleman, R.E., Colomer, R., Costa, L., Crino, L., Dirix, L., Gnant, M., Gralow, J., Hadji, P., Hortobagyi, G.N., Jonat, W., Lipton, A., Monnier, A., Paterson, A.H., Rizzoli, R., Saad, F. and Thurlimann, B. (2008) Guidance on the use of bisphosphonates in solid tumours: recommendations of an international expert panel. *Ann Oncol*, **19**, 420-432.

Adami, H.O., Malker, B., Holmberg, L., Persson, I. and Stone, B. (1986) The relation between survival and age at diagnosis in breast cancer. *N Engl J Med*, **315**, 559-563.

Anders, C.K., Fan, C., Parker, J.S., Carey, L.A., Blackwell, K.L., Klauber-Demore, N. and Perou, C.M. (2011) Breast carcinomas arising at a young age: unique biology or a surrogate for aggressive intrinsic subtypes? *J Clin Oncol*, **29**, e18-20.

Anders, C.K., Hsu, D.S., Broadwater, G., Acharya, C.R., Foekens, J.A., Zhang, Y., Wang, Y., Marcom, P.K., Marks, J.R., Febbo, P.G., Nevins, J.R., Potti, A. and Blackwell, K.L. (2008) Young age at diagnosis correlates with worse prognosis and defines a subset of breast cancers with shared patterns of gene expression. *J Clin Oncol*, **26**, 3324-3330.

Asselin-Labat, M.L., Shackleton, M., Stingl, J., Vaillant, F., Forrest, N.C., Eaves, C.J., Visvader, J.E. and Lindeman, G.J. (2006) Steroid hormone receptor status of mouse mammary stem cells. *J Natl Cancer Inst*, **98**, 1011-1014.

Asselin-Labat, M.L., Vaillant, F., Sheridan, J.M., Pal, B., Wu, D., Simpson, E.R., Yasuda, H., Smyth, G.K., Martin, T.J., Lindeman, G.J. and Visvader, J.E. (2010) Control of mammary stem cell function by steroid hormone signalling. *Nature*, **465**, 798-802.

Azim, H.A., Jr., Michiels, S., Bedard, P., Singhal, S.K., Criscitiello, C., Ignatiadis, M., Haibe-Kains, B., Piccart, M., Sotiriou, C. and Loi, S. (2012) Elucidating prognosis and biology of breast cancer arising in young women using gene expression profiling. *Clin Cancer Res*, **18**, 1241-1251.

Bamias, A., Kastritis, E., Bamia, C., Moulopoulos, L.A., Melakopoulos, I., Bozas, G., Koutsoukou, V., Gika, D., Anagnostopoulos, A., Papadimitriou, C., Terpos, E. and Dimopoulos, M.A. (2005) Osteonecrosis of the jaw in cancer after treatment with bisphosphonates: incidence and risk factors. *J Clin Oncol*, **23**, 8580-8587.

Baselga, J., Bradbury, I., Eidtmann, H., Di Cosimo, S., de Azambuja, E., Aura, C., Gomez, H., Dinh, P., Fauria, K., Van Dooren, V., Aktan, G., Goldhirsch, A., Chang, T.W., Horvath,

Z., Coccia-Portugal, M., Domont, J., Tseng, L.M., Kunz, G., Sohn, J.H., Semiglazov, V., Lerzo, G., Palacova, M., Probachai, V., Pusztai, L., Untch, M., Gelber, R.D. and Piccart-Gebhart, M. (2012) Lapatinib with trastuzumab for HER2-positive early breast cancer (NeoALTTO): a randomised, open-label, multicentre, phase 3 trial. *Lancet*, **379**, 633-640.

Baselga, J., Semiglazov, V., van Dam, P., Manikhas, A., Bellet, M., Mayordomo, J., Campone, M., Kubista, E., Greil, R., Bianchi, G., Steinseifer, J., Molloy, B., Tokaji, E., Gardner, H., Phillips, P., Stumm, M., Lane, H.A., Dixon, J.M., Jonat, W. and Rugo, H.S. (2009) Phase II randomized study of neoadjuvant everolimus plus letrozole compared with placebo plus letrozole in patients with estrogen receptor-positive breast cancer. *J Clin Oncol*, **27**, 2630-2637.

Bedard, P.L., Singhal, S.K., Ignatiadis, M., Bradbury, I., Haibe-Kains, B., Desmedt, C., Loi, S., Evans, D.B., Michiels, S., Dixon, J.M., Miller, W.R., Piccart, M.J. and Sotiriou, C. (2011) Low residual proliferation after short-term letrozole therapy is an early predictive marker of response in high proliferative ER-positive breast cancer. *Endocr Relat Cancer*, **18**, 721-730.

Bekker, P.J., Holloway, D.L., Rasmussen, A.S., Murphy, R., Martin, S.W., Leese, P.T., Holmes, G.B., Dunstan, C.R. and DePaoli, A.M. (2004) A single-dose placebo-controlled study of AMG 162, a fully human monoclonal antibody to RANKL, in postmenopausal women. *J Bone Miner Res*, **19**, 1059-1066.

Bharat, A., Aft, R.L., Gao, F. and Margenthaler, J.A. (2009) Patient and tumor characteristics associated with increased mortality in young women (< or =40 years) with breast cancer. *J Surg Oncol*, **100**, 248-251.

Brinton, L.A., Sherman, M.E., Carreon, J.D. and Anderson, W.F. (2008) Recent trends in breast cancer among younger women in the United States. *J Natl Cancer Inst*, **100**, 1643-1648.

Cancello, G., Maisonneuve, P., Rotmensz, N., Viale, G., Mastropasqua, M.G., Pruneri, G., Veronesi, P., Torrisi, R., Montagna, E., Luini, A., Intra, M., Gentilini, O., Ghisini, R., Goldhirsch, A. and Colleoni, M. (2010) Prognosis and adjuvant treatment effects in selected breast cancer subtypes of very young women (<35 years) with operable breast cancer. *Ann Oncol*, **21**, 1974-1981.

Canon, J.R., Roudier, M., Bryant, R., Morony, S., Stolina, M., Kostenuik, P.J. and Dougall, W.C. (2008) Inhibition of RANKL blocks skeletal tumor progression and improves survival

in a mouse model of breast cancer bone metastasis. *Clin Exp Metastasis*, **25**, 119-129.

Chung, M., Chang, H.R., Bland, K.I. and Wanebo, H.J. (1996) Younger women with breast carcinoma have a poorer prognosis than older women. *Cancer*, **77**, 97-103.

Coleman, R.E. (2006) Clinical features of metastatic bone disease and risk of skeletal morbidity. *Clin Cancer Res*, **12**, 6243s-6249s.

Denkert, C., Loibl, S., Noske, A., Roller, M., Muller, B.M., Komor, M., Budczies, J., Darb-Esfahani, S., Kronenwett, R., Hanusch, C., von Torne, C., Weichert, W., Engels, K., Solbach, C., Schrader, I., Dietel, M. and von Minckwitz, G. (2010) Tumor-associated lymphocytes as an independent predictor of response to neoadjuvant chemotherapy in breast cancer. *J Clin Oncol*, **28**, 105-113.

Desmedt, C., Haibe-Kains, B., Wirapati, P., Buyse, M., Larsimont, D., Bontempi, G., Delorenzi, M., Piccart, M. and Sotiriou, C. (2008) Biological processes associated with breast cancer clinical outcome depend on the molecular subtypes. *Clin Cancer Res*, **14**, 5158-5165.

Dimopoulos, M.A., Kastritis, E., Bamia, C., Melakopoulos, I., Gika, D., Roussou, M., Migkou, M., Eleftherakis-Papaiakovou, E., Christoulas, D., Terpos, E. and Bamias, A. (2009) Reduction of osteonecrosis of the jaw (ONJ) after implementation of preventive measures in patients with multiple myeloma treated with zoledronic acid. *Ann Oncol*, **20**, 117-120.

Dowsett, M., Nielsen, T.O., A'Hern, R., Bartlett, J., Coombes, R.C., Cuzick, J., Ellis, M., Henry, N.L., Hugh, J.C., Lively, T., McShane, L., Paik, S., Penault-Llorca, F., Prudkin, L., Regan, M., Salter, J., Sotiriou, C., Smith, I.E., Viale, G., Zujewski, J.A. and Hayes, D.F. (2011) Assessment of Ki67 in breast cancer: recommendations from the International Ki67 in Breast Cancer working group. *J Natl Cancer Inst*, **103**, 1656-1664.

Durie, B.G., Katz, M. and Crowley, J. (2005) Osteonecrosis of the jaw and bisphosphonates. *N Engl J Med*, **353**, 99-102; discussion 199-102.

El Saghir, N.S., Seoud, M., Khalil, M.K., Charafeddine, M., Salem, Z.K., Geara, F.B. and Shamseddine, A.I. (2006) Effects of young age at presentation on survival in breast cancer. *BMC Cancer*, **6**, 194.

Elliot, R., Kostenuik, P., Chen, C. and al., e. (2006) Denosumab is a selective inhibitor of human receptor activator of NF-κß ligand that blocks osteoclast formation in vitro and in vivo. Eur J Ca Suppl *Eur J Cancer Suppl*, **4**, 62.

Fizazi, K., Carducci, M., Smith, M., Damiao, R., Brown, J., Karsh, L., Milecki, P., Shore, N., Rader, M., Wang, H., Jiang, Q., Tadros, S., Dansey, R. and Goessl, C. (2011) Denosumab versus zoledronic acid for treatment of bone metastases in men with castration-resistant prostate cancer: a randomised, double-blind study. *Lancet*, **377**, 813-822.

Fizazi, K., Lipton, A., Mariette, X., Body, J.J., Rahim, Y., Gralow, J.R., Gao, G., Wu, L., Sohn, W. and Jun, S. (2009) Randomized phase II trial of denosumab in patients with bone metastases from prostate cancer, breast cancer, or other neoplasms after intravenous bisphosphonates. *J Clin Oncol*, **27**, 1564-1571.

Fredholm, H., Eaker, S., Frisell, J., Holmberg, L., Fredriksson, I. and Lindman, H. (2009) Breast cancer in young women: poor survival despite intensive treatment. *PLoS One*, **4**, e7695.

Gonzalez-Suarez, E., Jacob, A.P., Jones, J., Miller, R., Roudier-Meyer, M.P., Erwert, R., Pinkas, J., Branstetter, D. and Dougall, W.C. (2010) RANK ligand mediates progestin-induced mammary epithelial proliferation and carcinogenesis. *Nature*, **468**, 103-107.

Henry, D.H., Costa, L., Goldwasser, F., Hirsh, V., Hungria, V., Prausova, J., Scagliotti, G.V., Sleeboom, H., Spencer, A., Vadhan-Raj, S., von Moos, R., Willenbacher, W., Woll, P.J., Wang, J., Jiang, Q., Jun, S., Dansey, R. and Yeh, H. (2011) Randomized, double-blind study of denosumab versus zoledronic acid in the treatment of bone metastases in patients with advanced cancer (excluding breast and prostate cancer) or multiple myeloma. *J Clin Oncol*, **29**, 1125-1132.

ISBER. (2008) 2008 Beast Practices for Repositories. Collection, storage, retrieval and distribution of biological material for research. Cell Preservation technology Vol 6, Number 1.

Joshi, P.A., Jackson, H.W., Beristain, A.G., Di Grappa, M.A., Mote, P.A., Clarke, C.L., Stingl, J., Waterhouse, P.D. and Khokha, R. (2010) Progesterone induces adult mammary stem cell expansion. *Nature*, **465**, 803-807.

Khosla, S. (2001) Minireview: the OPG/RANKL/RANK system. *Endocrinology*, **142**, 5050-5055.

Kim, E.K., Noh, W.C., Han, W. and Noh, D.Y. (2011) Prognostic Significance of Young Age (<35 Years) by Subtype Based on ER, PR, and HER2 Status in Breast Cancer: A Nationwide Registry-Based Study. *World J Surg*, **37**, 693-699.

Kohno, N., Aogi, K., Minami, H., Nakamura, S., Asaga, T., Iino, Y., Watanabe, T., Goessl, C., Ohashi, Y. and Takashima, S. (2005) Zoledronic acid significantly reduces skeletal complications compared with placebo in Japanese women with bone metastases

from breast cancer: a randomized, placebo-controlled trial. *J Clin Oncol*, **23**, 3314-3321.

Kollias, J., Elston, C.W., Ellis, I.O., Robertson, J.F. and Blamey, R.W. (1997) Early-onset breast cancer--histopathological and prognostic considerations. *Br J Cancer*, **75**, 1318-1323.

Kostenuik, P.J., Nguyen, H.Q., McCabe, J., Warmington, K.S., Kurahara, C., Sun, N., Chen, C., Li, L., Cattley, R.C., Van, G., Scully, S., Elliott, R., Grisanti, M., Morony, S., Tan, H.L., Asuncion, F., Li, X., Ominsky, M.S., Stolina, M., Dwyer, D., Dougall, W.C., Hawkins, N., Boyle, W.J., Simonet, W.S. and Sullivan, J.K. (2009) Denosumab, a fully human monoclonal antibody to RANKL, inhibits bone resorption and increases BMD in knock-in mice that express chimeric (murine/human) RANKL. *J Bone Miner Res*, **24**, 182-195.

Kumagai, Y., Hasunuma, T. and Padhi, D. (2011) A randomized, double-blind, placebo-controlled, single-dose study to evaluate the safety, tolerability, pharmacokinetics and pharmacodynamics of denosumab administered subcutaneously to postmenopausal Japanese women. *Bone*, **49**, 1101-1107.

Lacey, D.L., Timms, E., Tan, H.L., Kelley, M.J., Dunstan, C.R., Burgess, T., Elliott, R., Colombero, A., Elliott, G., Scully, S., Hsu, H., Sullivan, J., Hawkins, N., Davy, E., Capparelli, C., Eli, A., Qian, Y.X., Kaufman, S., Sarosi, I., Shalhoub, V., Senaldi, G., Guo, J., Delaney, J. and Boyle, W.J. (1998) Osteoprotegerin ligand is a cytokine that regulates osteoclast differentiation and activation. *Cell*, **93**, 165-176.

Landi, M.T. and Caporaso, N. (1997) Sample collection, processing and storage. *IARC Sci Publ*, 223-236.

Lim, E., Vaillant, F., Wu, D., Forrest, N.C., Pal, B., Hart, A.H., Asselin-Labat, M.L., Gyorki, D.E., Ward, T., Partanen, A., Feleppa, F., Huschtscha, L.I., Thorne, H.J., Fox, S.B., Yan, M., French, J.D., Brown, M.A., Smyth, G.K., Visvader, J.E. and Lindeman, G.J. (2009) Aberrant luminal progenitors as the candidate target population for basal tumor development in BRCA1 mutation carriers. *Nat Med*, **15**, 907-913.

Lipton, A., Siena, S., Rader, M., Bilynskyy, B., Viniegra, M., Richardson, G., Beuzeboc, P., Clemens, M., Ke, C. and Jun, S. (2010) Comparison of denosumab vs. zoledronic acid (ZA) for treatment of bone metastases in advanced cancer patients: An integrated analysis of 3 pivotal trials. *Ann Oncol*, **21**, vii 380.

Lipton, A., Steger, G.G., Figueroa, J., Alvarado, C., Solal-Celigny, P., Body, J.J., de Boer, R., Berardi, R., Gascon, P., Tonkin, K.S., Coleman, R., Paterson, A.H., Peterson, M.C., Fan, M., Kinsey, A. and Jun, S. (2007) Randomized active-controlled phase II study of

denosumab efficacy and safety in patients with breast cancer-related bone metastases. *J Clin Oncol*, **25**, 4431-4437.

Marx, R.E. (2003) Pamidronate (Aredia) and zoledronate (Zometa) induced avascular necrosis of the jaws: a growing epidemic. *J Oral Maxillofac Surg*, **61**, 1115-1117.

McCall, M.N., Bolstad, B.M. and Irizarry, R.A. (2010) Frozen robust multiarray analysis (fRMA). *Biostatistics*, **11**, 242-253.

Migliorati, C.A. (2003) Bisphosphanates and oral cavity avascular bone necrosis. *J Clin Oncol*, **21**, 4253-4254.

Miller, W.R., Larionov, A., Renshaw, L., Anderson, T.J., Walker, J.R., Krause, A., Sing, T., Evans, D.B. and Dixon, J.M. (2009) Gene expression profiles differentiating between breast cancers clinically responsive or resistant to letrozole. *J Clin Oncol*, **27**, 1382-1387.

Morony, S., Capparelli, C., Sarosi, I., Lacey, D.L., Dunstan, C.R. and Kostenuik, P.J. (2001) Osteoprotegerin inhibits osteolysis and decreases skeletal tumor burden in syngeneic and nude mouse models of experimental bone metastasis. *Cancer Res*, **61**, 4432-4436.

Mundy, G.R. (1993) Cytokines and growth factors in the regulation of bone remodeling. *J Bone Miner Res*, **8 Suppl 2**, S505-510.

Ripamonti, C.I., Maniezzo, M., Campa, T., Fagnoni, E., Brunelli, C., Saibene, G., Bareggi, C., Ascani, L. and Cislaghi, E. (2009) Decreased occurrence of osteonecrosis of the jaw after implementation of dental preventive measures in solid tumour patients with bone metastases treated with bisphosphonates. The experience of the National Cancer Institute of Milan. *Ann Oncol*, **20**, 137-145.

Rojo, F., Najera, L., Lirola, J., Jimenez, J., Guzman, M., Sabadell, M.D., Baselga, J. and Ramon y Cajal, S. (2007) 4E-binding protein 1, a cell signaling hallmark in breast cancer that correlates with pathologic grade and prognosis. *Clin Cancer Res*, **13**, 81-89.

Roodman, G.D. (2004) Mechanisms of bone metastasis. *N Engl J Med*, **350**, 1655-1664.

Schramek, D., Leibbrandt, A., Sigl, V., Kenner, L., Pospisilik, J.A., Lee, H.J., Hanada, R., Joshi, P.A., Aliprantis, A., Glimcher, L., Pasparakis, M., Khokha, R., Ormandy, C.J., Widschwendter, M., Schett, G. and Penninger, J.M. (2010) Osteoclast differentiation factor RANKL controls development of progestin-driven mammary cancer. *Nature*, **468**, 98-102.

Sotiriou, C., Wirapati, P., Loi, S., Harris, A., Fox, S., Smeds, J., Nordgren, H., Farmer, P., Praz, V., Haibe-Kains, B., Desmedt, C., Larsimont, D., Cardoso, F., Peterse, H., Nuyten, D., Buyse, M., Van de Vijver, M.J., Bergh, J., Piccart, M. and Delorenzi, M. (2006) Gene

expression profiling in breast cancer: understanding the molecular basis of histologic grade to improve prognosis. *J Natl Cancer Inst*, **98**, 262-272.

Stopeck, A.T., Lipton, A., Body, J.J., Steger, G.G., Tonkin, K., de Boer, R.H., Lichinitser, M., Fujiwara, Y., Yardley, D.A., Viniegra, M., Fan, M., Jiang, Q., Dansey, R., Jun, S. and Braun, A. (2010) Denosumab compared with zoledronic acid for the treatment of bone metastases in patients with advanced breast cancer: a randomized, double-blind study. *J Clin Oncol*, **28**, 5132-5139.

Symmans, W.F., Hatzis, C., Sotiriou, C., Andre, F., Peintinger, F., Regitnig, P., Daxenbichler, G., Desmedt, C., Domont, J., Marth, C., Delaloge, S., Bauernhofer, T., Valero, V., Booser, D.J., Hortobagyi, G.N. and Pusztai, L. (2010) Genomic index of sensitivity to endocrine therapy for breast cancer. *J Clin Oncol*, **28**, 4111-4119.

Tabrizi, M.A., Tseng, C.M. and Roskos, L.K. (2006) Elimination mechanisms of therapeutic monoclonal antibodies. *Drug Discov Today*, **11**, 81-88.

Tan, W., Zhang, W., Strasner, A., Grivennikov, S., Cheng, J.Q., Hoffman, R.M. and Karin, M. (2011) Tumour-infiltrating regulatory T cells stimulate mammary cancer metastasis through RANKL-RANK signalling. *Nature*, **470**, 548-553.

Teitelbaum, S.L. and Ross, F.P. (2003) Genetic regulation of osteoclast development and function. *Nat Rev Genet*, **4**, 638-649.

Teschendorff, A.E., Miremadi, A., Pinder, S.E., Ellis, I.O. and Caldas, C. (2007) An immune response gene expression module identifies a good prognosis subtype in estrogen receptor negative breast cancer. *Genome Biol*, **8**, R157.

Thomas, D., Henshaw, R., Skubitz, K., Chawla, S., Staddon, A., Blay, J.Y., Roudier, M., Smith, J., Ye, Z., Sohn, W., Dansey, R. and Jun, S. (2010) Denosumab in patients with giant-cell tumour of bone: an open-label, phase 2 study. *Lancet Oncol*, **11**, 275-280.

Wang, W., Wang, E.Q. and Balthasar, J.P. (2008) Monoclonal antibody pharmacokinetics and pharmacodynamics. *Clin Pharmacol Ther*, **84**, 548-558.

Wardley, A., Davidson, N., Barrett-Lee, P., Hong, A., Mansi, J., Dodwell, D., Murphy, R., Mason, T. and Cameron, D. (2005) Zoledronic acid significantly improves pain scores and quality of life in breast cancer patients with bone metastases: a randomised, crossover study of community vs hospital bisphosphonate administration. *Br J Cancer*, **92**, 1869-1876.

XGEVA. (2011) Product monograph; XGEVA (denosumab) 120mg/1.7 mL solution for injection, Amgen

13. APPENDIX 1: ECOG PERFORMANCE STATUS

GRADE	ECOG
0	Fully active, able to carry on all pre-disease performance without restriction
1	Restricted in physically strenuous activity but ambulatory and able to carry out work of a light or sedentary nature, e.g., light house work, office work
2	Ambulatory and capable of all selfcare but unable to carry out any work activities. Up and about more than 50% of waking hours
3	Capable of only limited selfcare, confined to bed or chair more than 50% of waking hours
4	Completely disabled. Cannot carry on any selfcare. Totally confined to bed or chair
5	Dead

* As published in Am. J. Clin. Oncol.:

Oken, M.M., Creech, R.H., Tormey, D.C., Horton, J., Davis, T.E., McFadden, E.T., Carbone, P.P.: Toxicity And Response Criteria Of The Eastern Cooperative Oncology Group. Am J Clin Oncol 5:649-655, 1982.

14. APPENDIX 2: COMMON TERMINOLOGY CRITERIA FOR ADVERSE EVENTS

In the present study, AEs and/or adverse drug reactions will be recorded according to the Common Terminology Criteria for Adverse Events (CTCAE), version 4.0.

At the time this protocol was issued, the full CTCAE document was available on the NCI web site, at the following address: http://ctep.cancer.gov/reporting/ctc.html.

The EORTC Data Center web site http://www.eortc.be/ provides a link to the appropriate CTCAE web site. This link will be updated if the CTCAE address is changed.

Investigators who do not have access to Internet can contact the BrEAST Data Centre to receive a hard copy of this document by mail.
